# Diverse ERBB2/ERBB3 Activating Alterations and Coalterations Have Implications for HER2/3-Targeted Therapies across Solid Tumors

**DOI:** 10.1158/2767-9764.CRC-24-0620

**Published:** 2025-04-25

**Authors:** Dazhi Liu, Justin Jee, Alexander Drilon, Andreas M. Heilmann, Justin M. Allen, Alexa B. Schrock, Rachel B. Keller-Evans, Bob T. Li

**Affiliations:** 1 Memorial Sloan Kettering Cancer Center, New York, New York.; 2 Weill Cornell Medical College, New York, New York.; 3 Foundation Medicine, Inc., Cambridge, Massachusetts.

## Abstract

Although *ERBB2* (HER2) is an established oncogenic driver and therapeutic biomarker in several cancers, current drug approvals do not reflect the diverse spectrum of activating alterations across indications in which HER2-targeted therapies may yield clinical benefit. In most cancer types, HER2 status is defined by HER2 overexpression/amplification assessed by IHC and FISH, which do not provide genomic context. We sought to define the pan-tumor landscape of activating *ERBB2* and *ERBB3* genomic alterations detected by comprehensive genomic profiling (CGP). We queried institutional databases of solid tumor CGP, including 429,666 patients who underwent Foundation Medicine testing and 83,332 patients whose tumors were profiled using Memorial Sloan Kettering Integrated Mutation Profiling of Actionable Cancer Targets (MSK-IMPACT). We identified activating *ERBB2* and *ERBB3* alterations across solid tumor types, including many off-label for current HER2 drug approvals. Whereas non–small cell lung cancer represented the highest proportion of *ERBB2*-mutated (i.e., single-nucleotide variants and short insertions/deletions) cancers (19.0%), breast, colorectal, bladder, and gastroesophageal cancers combined accounted for 50.4% of *ERBB2-*mutated tumors. Within non–small cell lung cancer, 26% of activating mutations were not included in clinical trials that led to approval of the antibody–drug conjugate trastuzumab deruxtecan. We also present three clinical cases demonstrating clinical benefit from off-label use of HER2-targeted therapies. We identified substantial populations of patients with diverse *ERBB2*/*ERBB3* activating alterations, which represent unmet therapeutic needs. We demonstrate that CGP provides additional genomic information, inclusive of *ERBB2* amplification and mutation status together with potential resistance/response-modifying co-alterations, allowing for more nuanced HER2 status interpretation than is possible with IHC/FISH alone.

**Significance:**

CGP provides genomic context for HER2 status beyond the information provided by IHC and FISH, including detection of *ERBB2* mutations and co-alterations that may suggest sensitivity/resistance to HER2-directed therapies, and is therefore crucial for guiding treatment choice and understanding individual patient response.

## Introduction

Genomic profiling has identified *ERBB2* amplification and mutations, encoding overexpressed or hyperactivated HER2, and *ERBB3* (encoding HER3) alterations as drivers of human cancers (1). When *ERBB2* (HER2) is amplified or mutated, it becomes activated in a ligand-independent manner, driving activation of downstream pathways controlling the migration, proliferation, survival, and differentiation of cancer cells (1). Multiple HER2-targeted therapies have been approved to treat HER2 amplification and/or overexpression in breast cancer via different mechanisms of action, including monoclonal antibodies (mAb; e.g., trastuzumab), tyrosine kinase inhibitors (TKI, e.g., neratinib), and antibody–drug conjugates [ADC, e.g., trastuzumab emtansine and trastuzumab deruxtecan (T-DXd); ref. 2]. Trastuzumab and T-DXd are also approved for the treatment of HER2-amplified and/or -overexpressed gastric cancers (3). In the last 2 years, T-DXd was approved to treat non–small cell lung cancer (NSCLC) with select *ERBB2* mutations (4), and tucatinib plus trastuzumab was approved to treat HER2 amplification and/or overexpression in colorectal cancer (CRC) (5). Most recently, T-DXd received pan-tumor approval for unresectable or metastatic HER2-overexpressing (IHC 3+) solid tumors (6–8).

Recent trials investigating HER2-targeted therapies have demonstrated efficacy in solid tumors with *ERBB2* (HER2) alterations in indications which have not been approved by the U.S. FDA (9). Specifically, the lack of approved HER2-targeted therapies for non-NSCLC with *ERBB2* mutations in the absence of amplification/overexpression suggests an unmet clinical need for a substantial population of patients (10, 11). In addition, novel therapies—e.g., patritumab deruxtecan, a novel HER3-directed ADC that has exhibited *in vitro* activity against *ERBB3* mutations in breast cancer models (12)—will require exploration of informative biomarkers. In this study, we report the pan-tumor landscape of *ERBB2* and *ERBB3* activating alterations across a combined cohort of >500,000 patients with either Foundation Medicine or Memorial Sloan Kettering Integrated Mutation Profiling of Actionable Cancer Targets (MSK-IMPACT) comprehensive genomic profiling (CGP). Our findings provide important insight into unmet therapeutic needs for patients with cancers harboring *ERBB2*/*ERBB3* alterations, which is essential for a new paradigm of pan-tumor HER2-targeted therapy.

## Materials and Methods

### Foundation Medicine genomic cohort

We queried an institutional database of pan-solid tumor CGP performed during the course of routine clinical care between August 2014 and May 2022. For tissue biopsy samples, FoundationOneCDx (F1CDx) was performed on hybrid-capture, adapter ligation–based libraries using DNA extracted from formalin-fixed paraffin-embedded (FFPE) tumor and interrogated for alterations in 324 or 404 cancer-associated genes and selected introns from 31 or 34 genes that are frequently rearranged in cancer (see Supplementary Materials and Methods; refs. 13, 14). For liquid biopsy samples, FoundationOneLiquid CDx (F1LCDx) was performed on hybrid-capture, adapter ligation–based libraries using cell-free DNA extracted from peripheral blood plasma samples interrogated for alterations in 324 cancer-associated genes (15, 16). CGP testing was performed in a Clinical Laboratory Improvement Amendments–certified, College of American Pathologists–accredited, New York State–approved laboratory (Foundation Medicine, Inc.). Approval for this study, including a waiver of written informed consent and a Health Insurance Portability and Accountability Act (HIPAA) waiver of authorization, was obtained from the Western Institutional Review Board (Protocol No. 20152817).

### Co-alteration analysis

The prevalence of known or likely oncogenic genomic alterations in *ERBB2*-mutated (*ERBB2*mut)/nonamplified tumors versus *ERBB2* wild-type (WT, i.e., nonmutated and nonamplified) tumors was compared. Only genes altered in ≥50 tumors within the overall cohort and genes profiled across all tissue CGP assay versions were included. The Fisher exact test was performed to assess patterns of co-occurrence/mutual exclusivity between *ERBB2* and other genes and corrections for multiple testing were made using the Benjamini–Hochberg FDR method.

### Paired tissue/liquid CGP analysis

A subanalysis of the tissue cohort looked at patients with matched liquid biopsy requiring that (i) the cancer diagnosis associated with both biopsies matched and (ii) the liquid biopsy specimen was collected 90 days prior up to 2 years after the collection of the tissue biopsy specimen (*N* = 1,922). If a patient had multiple liquid biopsies, the specimen collected closest to the collection date of the matched tissue biopsy was used. The level of ctDNA shed in liquid biopsies was quantified by calculating an investigational measure that merges two methods for estimation of ctDNA tumor fraction (ctDNA TF; ref. 17). A threshold of ctDNA TF ≥ 1% was used, as this cutoff has been shown to have utility in informing negative biopsy results (18). An exploratory cutoff of ctDNA TF ≥ 10% was also used. Ninety-five percent confidence intervals for positive percent agreement/negative predictive value were calculated in Python (version 3.9.12; RRID: SCR_008394) using the Wilson Continuity Corrected Method (“wilsoncc”) from the statistical functions module of SciPy (version 1.7.3; RRID: SCR_008058).

### Memorial Sloan Kettering Cancer Center clinicogenomic cohort

Patients with solid tumors harboring activating *ERBB2* mutations were identified utilizing the institution-wide next-generation sequencing (NGS) program MSK-IMPACT. Activating/likely activating alterations were classified using OncoKB (RRID: SCR_014782; refs. 19, 20). Clinical data, including baseline demographics, tumor characteristics, and treatment patterns, were analyzed using data extracted from electronic health records.

### Other statistical considerations

Statistical testing was performed using R (version 4.2.1; RRID: SCR_001905), unless otherwise specified. For the clinicogenomic feature cohort comparison, Fisher exact and χ^2^ tests were used, as appropriate, to assess differences in clinical features between the cohorts, and FDR corrections were made using the Benjamini–Hochberg method to correct *P* values for multiple tests.

### Data availability

The authors declare that all relevant aggregate data supporting the findings of this study are available within the article and its supplementary information files. The data that support the findings of this study originated from Foundation Medicine, Inc. In accordance with the Health Insurance Portability and Accountability Act, we do not have Institutional Review Board approval or patient consent to share individualized patient genomic data, which contain potentially identifying or sensitive patient information and cannot be reported in a public data repository. Foundation Medicine is committed to collaborative data analysis and has well-established and widely used mechanisms by which qualified researchers can query our core genomic database of >900,000 deidentified sequenced cancers. Interested academic researchers can submit a proposal to the Foundation Medicine Data Collaborations Committee. More information and mechanisms for data access can be obtained by contacting the corresponding author or the Foundation Medicine Data Governance Council at data.governance.council@foundationmedicine.com.

## Results

### Pan-tumor landscape of *ERBB2/ERBB3* activating alterations

**Figure 1:**
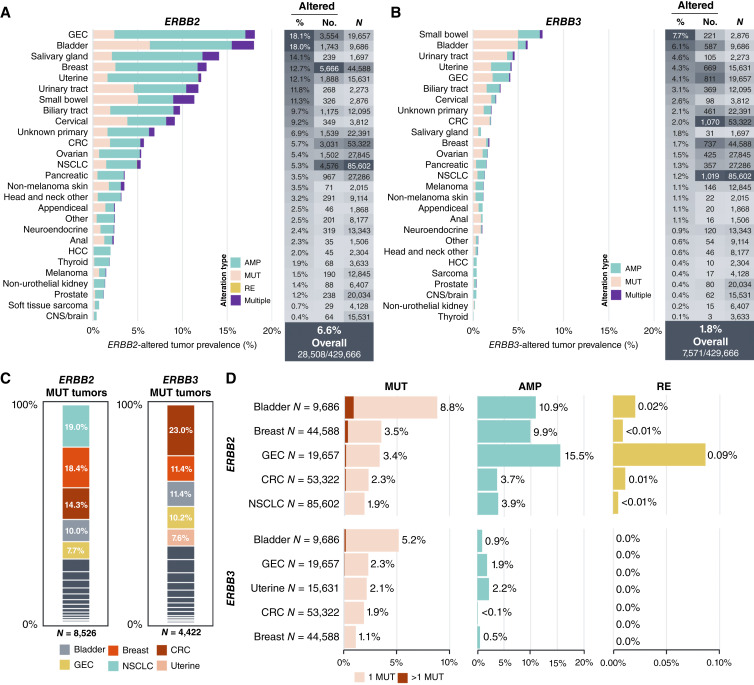
Pan-tumor landscape of *ERBB2/ERBB3* activating alterations detected via Foundation Medicine tissue comprehensive genomic profiling. Prevalence of (**A**) *ERBB2* and (**B**) *ERBB3* activating alterations (known or likely functional significance) across solid tumors. “Multiple” includes patients with AMP + MUT, AMP + RE, MUT + RE, and AMP + MUT + RE and patients with >1 MUT. **C,** Cancer type distribution of *ERBB2* (left) and *ERBB3* (right) MUT tumors. **D,** Prevalence of *ERBB2* and *ERBB3* alteration types in five major *ERBB2* or *ERBB3* MUT cancer types, respectively. Note that AMP/MUT/RE prevalence across plots are not additive due to tumors with multiple alterations. AMP, amplification; CNS, central nervous system; CRC, colorectal cancer; GEC, gastroesophageal cancer; HCC, hepatocellular carcinoma; MUT, mutation (single-nucleotide variants and insertions/deletions); NSCLC, non-small cell lung cancer; RE, rearrangement.

We assessed the presence of activating *ERBB2* and *ERBB3* alterations across 429,666 solid tumors profiled using F1CDx. Alterations considered to be activating in *ERBB2*/*ERBB3* included copy-number amplifications (AMP), mutations (MUT) of known or likely functional significance, and rare *ERBB2* intragenic rearrangements (RE) resulting in loss of exon 16 (Ex16Alt). *ERBB2* activating alterations were detected in 6.6% (*n* = 28,508) of tumors in the overall cohort and were most prevalent in gastroesophageal carcinoma (GEC; 18.1%), bladder (18.0%), salivary gland (14.1%), breast (12.7%), and uterine (12.1%) cancers (Fig. 1A). *ERBB3* activating alterations were detected in 1.8% (*n* = 7,571) of tumors in the overall cohort and were most prevalent in small bowel (7.7%), bladder (6.1%), urinary tract (4.6%), uterine (4.3%), and GEC (4.1%) cancers (Fig. 1B).

*ERBB2* amplification was most prevalent in GEC (15.5%), salivary gland (11.8%), bladder (10.9%), uterine (10.3%), and breast (9.9%) cancers, whereas *ERBB3* amplification was most prevalent in small bowel (2.4%), uterine (2.2%), GEC (1.9%), biliary tract (1.5%), and ovarian (1.2%) cancers (Fig. 1A and B). The median *ERBB2* amplification ratio (amp ratio, i.e., ratio of the modeled gene copy number to sample ploidy) ranged from 1.78 to 12.52 across tumor types (Q1 = 1.55–5.72, Q3 = 2.23–28.31), with salivary gland (median 12.52, IQR = 5.72–23.12), GEC (median 7.00, IQR = 2.83–27.63), breast (median 6.99, IQR = 2.45–15.46), colorectal (median 6.37, IQR = 2.23–28.31), and non-melanoma skin (median 6.14, IQR = 2.86–16.81) cancers exhibiting the highest median amp ratio across tumor types (Supplementary Fig. S1A). The median *ERBB3* amp ratio ranged from 2.15 to 3.93 (Q1 = 2.08–3.03, Q3 = 2.15–6.09; Supplementary Fig. S1B). *ERBB2* mutations were most prevalent in bladder (8.8%), small bowel (7.4%), urinary tract (5.9%), cervical (4.8%), and salivary gland (3.9%) cancers, whereas *ERBB3* mutations were most prevalent in small bowel (5.3%), bladder (5.2%), urinary tract (4.0%), GEC (2.3%), and uterine (2.1%) cancers (Fig. 1A and B). We selected the five most frequent tumor types in the *ERBB2*mut tumor cohort—NSCLC, breast cancer, CRC, bladder cancer, and GEC—as the subject of more focused analysis throughout the remainder of the study (Fig. 1C), with the exception of *ERBB3* mutation–specific analyses for which we focused on the most frequent tumor types in the *ERBB3-*mutated (*ERBB3*mut) tumor cohort (CRC, breast, bladder, GEC, and uterine cancers). The prevalence of *ERBB2* activating alteration types varied significantly across these cancer types (AMP: 3.7%–15.5%, MUT: 1.9%–8.8%, and RE: <0.01–0.09%), as did the prevalence of *ERBB3* activating alterations (AMP: <0.1%–2.2% and MUT: 1.1%–5.2%; Fig. 1D), and frequencies also varied by histology within a given tumor type (Supplementary Tables S1 and S2).

We compared the demographics and genomic characteristics of WT versus altered (ALT) *ERBB2* (*ERBB2*wt/alt) and *ERBB3* (*ERBB3*wt/alt) solid tumors (Table 1). Clinical features, including sex and genomic ancestry, were similar between the WT and ALT populations. Whereas difference in median age was statistically significant between both the *ERBB2* and *ERBB3* WT/ALT populations (*P* < 0.001 for both), this may be due to the large cohort size rather than due to clinically significant biological differences. A higher proportion of young-onset disease (<50 years) was observed in the *ERBB2*alt versus *ERBB2*wt population (16.3% vs. 13.9%, *P* < 0.001), and the inverse in the *ERBB3*alt versus *ERBB3*wt population (11.8% vs. 19.2%, *P* < 0.001). *ERBB3* alterations were associated with high microsatellite instability (MSI-H: 10.6% vs. 4.5%, *P* < 0.001), along with elevated tumor mutational burden [TMB; median 4.4 vs. 3.5 mutations per Mb (Mut/Mb), *P* < 0.001] and TMB-H (≥10 Mut/Mb: 25.1% vs. 11.3%; ≥20 Mut/Mb: 15.9% vs. 5.2%, *P* < 0.001 for both comparisons). Whereas *ERBB2* alterations were statistically associated with a higher median TMB (4.4 vs. 3.8 Mut/Mb, *P* < 0.001), the TMB ≥10 cutoff was not significantly different between the cohorts (20.7% vs. 20.3%; *P* = 0.11).

**Table 1:** Clinicogenomic characteristics of Foundation Medicine tissue comprehensive genomic profiling cohort stratified by *ERBB2*/*ERBB3* alteration status

Characteristic	*ERBB2* WT+ALT	*ERBB2* WT	*ERBB2* ALT	*P* value*	*ERBB3* WT+ALT	*ERBB3* WT	*ERBB3* ALT	*P* value
*N*	212,855	194,285	18,570	—	142,884	139,010	3,874	—
Age at Bx (years)								
Median (IQR)	64 (55, 72)	64 (55, 72)	63 (54, 71)	<0.001	62 (52, 70)	62 (52, 70)	65 (56, 73)	<0.001
Age <50 years, *N* (%)	29,944 (14.1)	26,920 (13.9)	3,024 (16.3)	<0.001	26,969 (19.0)	265,16 (19.2)	453 (11.8)	<0.001
Sex, *N* (%)				0.496				<0.001
Female	118,784 (55.8)	108,465 (55.8)	10,319 (55.6)	—	91,389 (64.0)	89,172 (64.1)	2,217 (57.2)	—
Male	94,071 (44.2)	85,820 (44.2)	8,251 (44.4)	—	51,495 (36.0)	49,838 (35.9)	1,657 (42.8)	—
Cancer type, *N* (%)				<0.001				<0.001
NSCLC	85,602 (40.2)	81,026 (41.7)	4,576 (24.6)	—	—	—	—	—
Breast	44,588 (20.9)	38,922 (20.0)	5,666 (30.5)	—	44,588 (31.2)	43,851 (31.5)	737 (19.0)	—
CRC	53,322 (25.1)	50,291 (25.9)	3,031 (16.3)	—	53,322 (37.3)	52,252 (37.6)	1,070 (27.6)	—
Bladder	9,686 (4.6)	7,943 (4.1)	1,743 (9.4)	—	9,686 (6.8)	9,099 (6.5)	587 (15.2)	—
GEC	19,657 (9.2)	16,103 (8.3)	3,554 (19.1)	—	19,657 (13.8)	18,846 (13.6)	811 (20.9)	—
Uterine	—	—	—	—	15,631 (10.9)	14,962 (10.8)	669 (17.3)	—
Genomic ancestry^a^, *N* (%)				0.009				0.219
AFR	23,444 (11.0)	21,490 (11.1)	1,954 (10.5)	—	17,444 (12.2)	16,992 (12.2)	452 (11.7)	—
AMR	16,427 (7.7)	14,929 (7.7)	1,498 (8.1)	—	13,574 (9.5)	13,239 (9.5)	335 (8.6)	—
EAS	8,292 (3.9)	7,506 (3.9)	786 (4.2)	—	5,329 (3.7)	5,188 (3.7)	141 (3.6)	—
EUR	160,012 (75.2)	146,088 (75.2)	13,924 (75.0)	—	103,259 (72.3)	100,408 (72.2)	2,851 (73.6)	—
SAS	1,789 (0.8)	1,618 (0.8)	171 (0.9)	—	1,459 (1.0)	1,411 (1.0)	48 (1.2)	—
Unknown	2,891 (1.4)	2,654 (1.4)	237 (1.3)	—	1,819 (1.3)	1,772 (1.3)	47 (1.2)	—
MSI status, *N* (%)				0.229				<0.001
MSI-H	4,337 (2.0)	3,958 (2.0)	379 (2.0)	—	6,653 (4.7)	6,243 (4.5)	410 (10.6)	—
MSS	200,595 (94.2)	183,139 (94.3)	17,456 (94.0)	—	130,310 (91.2)	127,023 (91.4)	3,287 (84.8)	—
Unknown/ambiguous	7,923 (3.7)	7,188 (3.7)	735 (4.0)	—	5,921 (4.1)	5,744 (4.1)	177 (4.6)	—
TMB (Mut/Mb)								
Median (IQR)	4.3 (2.5, 7.8)	3.8 (2.5, 7.8)	4.4 (2.5, 8.7)	<0.001	3.8 (1.7, 6.1)	3.5 (1.7, 6.1)	4.4 (2.5, 10.0)	<0.001
TMB ≥10	43,213 (20.3)	39,361 (20.3)	3,852 (20.7)	0.11	16,651 (11.7)	15,680 (11.3)	971 (25.1)	<0.001
TMB ≥20	15,126 (7.1)	13,578 (7.0)	1,548 (8.3)	<0.001	7,789 (5.5)	7,172 (5.2)	617 (15.9)	<0.001

*P* values are FDR-corrected and reflect comparison between *ERBB2/3* WT and *ERBB2/3* ALT populations, respectively.

Abbreviations: CRC, colorectal cancer; GEC, gastroesophageal cancer; MSI-H, microsatellite instability-high; MSS, microsatellite stable; NSCLC, non-small cell lung cancer; TMB, tumor mutational burden.

a Genomic Ancestry Reflects 1000 Genomes Project (RRID: SCR_006828) Super Populations: AFR, African; AMR, Admixed American; EAS, East Asian; EUR, European; SAS, South Asian.

### *ERBB2/ERBB3* activating mutations in select cancers

**Figure 2:**
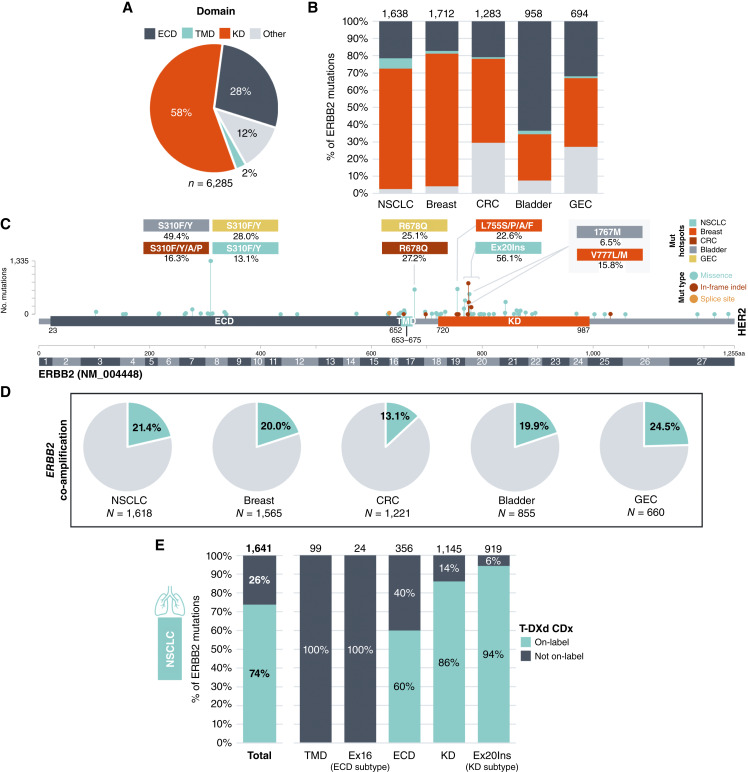
*ERBB2* activating mutations in select cancers. Distribution of *ERBB2* mutations across key HER2 protein domains (**A**) in the combined cohort of five major *ERBB2* MUT cancer types and (**B**) within each cancer type. The number of mutations is indicated. **C,** Lollipop plot mapping *ERBB2* mutations across the HER2 protein in select cancer types. The two most frequent codon hotspots for each cancer type are labeled; % represents the proportion of observed mutations at a particular codon in the respective cancer type. **D,** Rate of *ERBB2* co-amplification with *ERBB2* activating mutations in select cancer types. The number of *ERBB2* MUT tumors is indicated. **E, ***ERBB2* activating mutations detected in tissue CGP and reported by Foundation Medicine were compared with the list of mutations included in the DESTINYLung-01 trial. Matching mutations were considered “on-label,” and nonmatching mutations were considered “not on-label” for T-DXd in NSCLC. AA, amino acid; CDx, companion diagnostic; CRC, colorectal cancer; ECD, extracellular domain; GEC, gastroesophageal cancer; KD, kinase domain; MUT, mutation (single-nucleotide variants and insertions/deletions); NSCLC, non-small cell lung cancer; TMD, transmembrane domain; T-DXd, trastuzumab deruxtecan.

Among five major *ERBB2*mut tumor types, the largest proportion of mutations localized to the kinase domain (KD; 58%), followed by the extracellular domain (ECD; 28%) and other regions of the protein (12%), with rare mutations in the transmembrane domain (TMD; 2%; Fig. 2A). In contrast, *ERBB3* mutations were most commonly seen in the ECD (85% vs. 15% in the KD and <1% in other regions; Supplementary Fig. S2A). Missense substitutions predominated in both *ERBB2* and *ERBB3*. However, in-frame deletions were recurrent activating events in *ERBB2*, concentrated in exons 18, 19, and 20. Whereas *ERBB2* KD mutations predominated pan-tumor, the *ERBB2* mutational profile varied significantly between cancer types (Fig. 2B; Supplementary Fig. S3). NSCLC and breast cancer were enriched for KD mutations (69.9% and 77.0% of *ERBB2* mutations, respectively), whereas bladder cancer was dominated by ECD mutations (63.6%). CRC and GEC both harbored large proportions of mutations in non-ECD/non-KD regions (29.4% and 27.1%, respectively), largely driven by a high incidence of R678Q mutations. NSCLC harbored a markedly high incidence of TMD mutations (6.0% of *ERBB2* mutations compared with 1.0%–2.1% in other cancers). *ERBB3* mutational profiles were less variable across cancer types. However, breast cancer exhibited a divergently high proportion of KD mutations (53.1% of mutations compared with 2.9%–12.7% in other cancers; Supplementary Figs. S2B and S4).

Although there were shared *ERBB2* mutational hotspots across cancers (e.g., S310), certain mutation sites were uniquely enriched in specific contexts (Fig. 2C; Supplementary Fig. S3). Exon 20 insertions (Ex20Ins) were the dominant mutation type in NSCLC (56.1% of mutations), whereas L755 and V777 were hotspots in breast cancer (a combined 38.4% of *ERBB2* mutations). *ERBB3* mutational hotspots likewise varied across cancer types (Supplementary Figs. S2C and S4). Rare *ERBB2* activating alteration classes included TMD mutations (0.08% prevalence across select cancers; Supplementary Fig. S5A) and Ex16Alt (0.04% prevalence; Supplementary Fig. S5B) either through intragenic deletion (Ex16Del) or splice-site mutations (Ex16Splice). *ERBB2* activating mutations across all domains were largely clonal (ranging from 73.1%–92.0% of *ERBB2* mutations across cancer types), indicating they are often likely truncal in the tumor types studied (Supplementary Fig. S6). However, patterns of clonality did differ between tumor types. For example, GEC showed an especially high proportion of predicted subclonal *ERBB2* mutations across domains (25.2% ECD, 42.9% TMD, 26.0% KD, and 29.8% other) and a sizable proportion of ECD mutations in bladder cancer (23.8%) also seemed to be subclonal.

*ERBB2* co-amplification was observed in 13.1% to 24.5% of tumors harboring *ERBB2* activating mutations, suggesting that mutations can serve as the primary genomic mechanism of *ERBB2* activation in multiple tumor types (Fig. 2D). Ex16Alt co-occurred more frequently with *ERBB2* amplification (57.9% overall), with the majority of Ex16Del co-occurring [87.1% (27/31) vs. 37.2% (16/43) of Ex16Splice; Supplementary Fig. S5B).

We also explored the correlation between *ERBB2*mut status and HER2 IHC in a subset of *ERBB2*mut breast cancers for which IHC data were available (*n* = 16; Supplementary Fig. S7). HER2+ was reported for three tumors: an IHC 3+ tumor harboring *ERBB2* V777L with *ERBB2* co-amplification, an IHC 3+ tumor with D769Y + G776V and co-amplification, and an IHC 2+/FISH+ tumor with an Ex19Del (L755_T759del) which was non-amplified. *ERBB2* co-amplification was seen in three additional cases: an IHC 1+ tumor harboring an Ex20Ins (P780_Y781insGSP), an IHC 1+ tumor with G776V, and an IHC 2+/FISH- tumor with V777L. Notably, the degree of *ERBB2* amplification for the IHC 1+ samples was relatively low with amp ratios 2.15 (w/Ex20Ins) and 1.82 (w/G776V), respectively. Of the remaining 10 *ERBB2*mut cases which were all *ERBB2* non-amplified, six tumors were HER2-low [IHC 1+ (*n* = 5), IHC 2+ absent confirmatory FISH (*n* = 1)] and harbored a range of *ERBB2* mutations [Ex20Ins (A775_G776insYVMA), L755S (*n* = 2), and D769Y (*n* = 2), C630Y].

We compared activating mutations detected and reported using F1CDx to the list of eligible mutations included in the DESTINY-Lung01 trial (4). This trial informed the CDx label for the ADC T-DXd, which is FDA-approved for the treatment of unresectable or metastatic NSCLC harboring *ERBB2* mutations. In our study, 26% of activating/likely activating *ERBB2* mutations were not included in the CDx label, including 100% of TMD mutations (*n* = 99), 40% of ECD mutations [141/355, including 100% of Ex16Alt (*n* = 24)], and 14% of KD mutations [162/1,145, including 6% of Ex20Ins (55/919); Fig. 2E].

### Co-alteration landscape of *ERBB2*mut tumors in select cancers

We compared the prevalence of oncogenic co-alterations in *ERBB2*mut (excluding samples with *ERBB2* co-amplification) versus *ERBB2*wt (non-mutated and non-amplified) tumors among the five major *ERBB2*mut tumor types (Supplementary Fig. S8). *ERBB2* mutations tended to be mutually exclusive (*P* < 0.05, OR < 1) with other RTK/MAPK pathway driver oncogenes across cancer types, e.g., *EGFR*, *KRAS*, *ALK*, *MET*, *BRAF*, and *RET* in NSCLC (Supplementary Fig. S8A), *FGFR1* in breast cancer (Supplementary Fig. S8B), *BRAF* in CRC (Supplementary Fig. S8C), *FGFR3* in bladder cancer (Supplementary Fig. S8D), and *MYC* in GEC (Supplementary Fig. S8E), consistent with a driver role for *ERBB2* in these tumors. Whereas *KRAS* alterations and *ERBB2* mutations were shown to co-occur in CRC statistically, the percent difference was marginal (55.5% in *ERBB2*mut vs. 50.5% in *ERBB2*wt; Supplementary Fig. S8C). *NTRK3* alterations were also enriched in *ERBB2*mut CRC, but this association lost significance when limiting to the subset of microsatellite stable (MSS) tumors (Supplementary Fig. S8C and S8F), consistent with *NTRK* fusions having been previously associated with MSI-H CRC (21, 22). We observed 10 of 15 (66.7%) *NTRK3* fusions in the context of MSI-H *ERBB2*wt CRC, 3 of 15 (20.0%) in the context of MSI-H *ERBB2*mut CRC, and 2 of 15 (13.3%) in the context of MSS *ERBB2*wt CRC. *TP53* alterations tended to be mutually exclusive with *ERBB2* mutations across multiple cancer types. *ERBB3* alterations were found to co-occur with *ERBB2* mutations in all cancer types assessed except for NSCLC. Whereas enrichment of *ERBB3* co-alteration was maintained in MSS GEC (10.8% MUT vs. 3.6% WT; *P* < 0.001), this enrichment became non-significant in MSS CRC (3.1% MUT vs. 1.7% WT; *P* = 0.07), suggesting that *ERBB3* co-alteration could be MSI/TMB-associated in some cancers (Supplementary Fig. S8F and S8G and consistent with Table 1). Other interesting observations included the mutual exclusivity of *KEAP1* and *STK11* in NSCLC (Supplementary Fig. S8A) and enrichment for *CDH1*, *PIK3CA*, and *ARID1A* along with mutual exclusivity with *BRCA1/2*, *PTEN*, and *ESR1* in breast cancer (Supplementary Fig. S8B). The *ERBB2*/*CDH1*/*ESR1* observations were consistent with prior observations in metastatic breast cancer in which acquired *ERBB2* and *ESR1* resistance mutations were mutually exclusive, with *ERBB2* resistance mutations co-occurring with *CDH1* loss-of-function (LOF) mutations, suggesting enrichment in lobular breast cancers (23). Whereas enrichment of several WNT pathway–associated genes (*RNF43*, *CTNNB1*, and *APC*) was seen in GEC (Supplementary Fig. S8E), the *RNF43* and *APC* associations lost significance when limiting to the subset of MSS tumors (Supplementary Fig. S8G).

Closer examination of co-mutation patterns for *ERBB2* TMD mutations in NSCLC revealed divergent co-occurrence or mutual exclusivity with *EGFR* or *ERBB3* TMD mutations based on the specific *ERBB2* TMD allele. The most highly recurrent TMD mutations in NSCLC were V659E (62.2%, 61/98 TMD mutations), G660D (16.3%, 16/98), and V659D (12.2%, 12/98). Co-occurring *ERBB3* TMD (I649R) and *EGFR* TMD (G625R) mutations were found in 15.3% (15/98) and 3.1% (3/98) of TMD-mutated tumors, respectively. Strikingly, these co-occurring *EGFR*/*ERBB3* TMD mutations were found in association with V > D (83.3%, 10/12) or G > D (50.0%, 8/16) *ERBB2* TMD mutations but were absent in cases with V > E changes (0.0%, 0/61; Supplementary Fig. S9).

**Figure 3:**
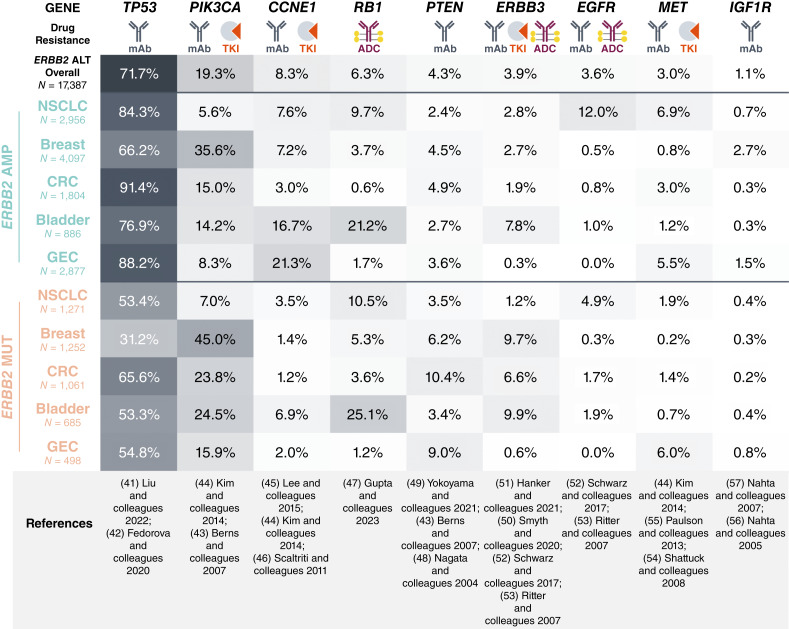
HER2-directed therapy response-modifying alterations in select cancers. Co-occurrence of alterations that have been reported to impact response to HER2-directed therapies (mAb, TKI, and ADC) in tumors with *ERBB2* amplification or *ERBB2* activating mutations, including *TP53* MUT, *PIK3CA* MUT, *CCNE1* AMP, *RB1* MUT/DEL, *PTEN* MUT/DEL, *ERBB3* AMP/MUT, *EGFR* MUT, *MET* AMP/Ex14 MUT, and *IGF1R* AMP. Patients with multiple *ERBB2* alteration types (e.g., AMP + MUT) were excluded from the analysis. ADC, antibody-drug conjugate; AMP, amplification; DEL, deep deletion; CRC, colorectal cancer; GEC, gastroesophageal cancer; mAB, monoclonal antibody; MUT, mutation (single-nucleotide variants and insertions/deletions); NSCLC, non-small cell lung cancer; TKI, tyrosine kinase inhibitor.

We also assessed the prevalence of alterations that have been reported to impact response to HER2-directed therapies (mAb, TKI, and ADC) in *ERBB2*-amplified (*ERBB2*amp) versus *ERBB2*mut tumors across select cancer types (Fig. 3; Supplementary Fig. S10). *TP53* mutations were, unsurprisingly, common across cancer types (71.7% overall across *ERBB2*alt tumors among the five major *ERBB2*mut tumor types) but co-occurred more frequently with *ERBB2* amplification than with *ERBB2* mutation (66.2%–91.4% vs. 31.2%–65.6%, respectively). *PIK3CA* mutations were detected at a high frequency in breast cancer and were more commonly found in *ERBB2*mut than in *ERBB2*amp tumors (45.0% vs. 35.6%). *CCNE1* amplification was observed frequently in *ERBB2*amp GEC (21.3%) and bladder cancer (16.7%) and less commonly in other tumors. *RB1* loss (inclusive of LOF mutations and deep deletions) was seen at similar frequencies in both *ERBB2*amp and *ERBB2*mut bladder cancer (21.2% and 25.1%, respectively) and NSCLC (9.7% and 10.5%, respectively) and less frequently in other tumor types. *PTEN* loss (inclusive of LOF mutations and deep deletions) was most prevalent in *ERBB2*mut CRC (10.4%) and GEC (9.0%) and generally less prevalent in *ERBB2*amp tumors. *ERBB3* coamplification/comutation was seen in *ERBB2*mut and *ERBB2*amp bladder cancer (9.9% and 7.8%, respectively), in *ERBB2*mut breast cancer (9.7%), and in *ERBB2*mut CRC (6.6%) but was more rare in other tumors. *EGFR* comutation was more common in *ERBB2*amp than in *ERBB2*mut NSCLC (12.0% vs. 4.9%) and infrequent in other tumor types. *MET* activation (inclusive of amplification and *MET*Ex14 mutations) was observed in 6.9% of *ERBB2*amp NSCLC and showed comparable prevalence in GEC between *ERBB2*amp and *ERBB2*mut tumors (5.5% and 6.0%, respectively), being less common in other tumor types. *IGF1R* amplification was rare (1.1% across *ERBB2*alt tumors) with a comparatively high prevalence in *ERBB2*amp breast cancer (2.7%).

### Detection of *ERBB2* activating alterations using liquid CGP in select cancers

**Figure 4:**
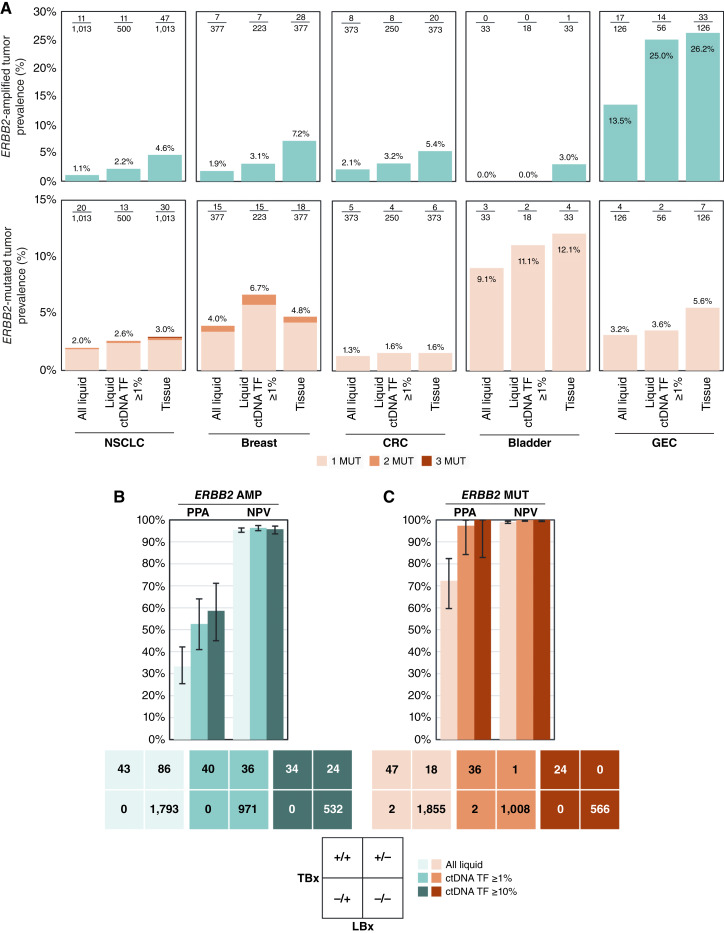
Detection of *ERBB2* activating alterations in select cancers via Foundation Medicine liquid comprehensive genomic profiling. **A,** Prevalence comparison of *ERBB2* amplification (top) and *ERBB2* mutations (bottom) detected in patient-matched tissue and liquid CGP across select cancer types in all matched liquid samples (*N* = 1,922) and only in matched liquid samples with elevated ctDNA TF (≥1%; *n* = 1,047). The number of samples in each group is indicated. PPA and NPV for liquid biopsy detection of (**B**) *ERBB2* amplification and (**C**) *ERBB2* mutations in select cancer types combined. Ninety-five percent confidence interval (CI; Wilson Continuity Corrected Method) are shown. AMP, amplification; CRC, colorectal cancer; GEC, gastroesophageal cancer; LBx, liquid biopsy; MUT, mutation (single-nucleotide variants and insertions/deletions); NSCLC, non-small cell lung cancer; NPV, negative predictive value; PPA, positive percent agreement; TBx, tissue biopsy.

We compared the prevalence of *ERBB2* alterations detected in patient-matched tissue and liquid biopsies in select cancers (Fig. 4A). Sensitivity for detecting *ERBB2* amplification was lower than for detecting *ERBB2* mutations across all cancers analyzed (33.3% vs. 72.3%, *N* = 1,922). However, sensitivity for detection of both amplification and mutations was improved in liquid samples with elevated ctDNA TF ≥ 1% (*n* = 1,047) and further still with ctDNA TF ≥ 10% (*n* = 590), increasing to 52.6% and 58.6% for amplification and 97.3% and 100% for mutations, respectively (Fig. 4B and C). The negative predictive value for detection of *ERBB2* amplifications and mutations in all liquid samples was 95.4% and 99.0%, respectively, improving to 96.4% and 99.9% when ctDNA TF was ≥1% and 95.7% and 100% when ctDNA TF was ≥10% (Fig. 4B and C).

### Clinical experience targeting uncommon *ERBB2* mutations in NSCLC/mutations in cancers beyond NSCLC

**Figure 5:**
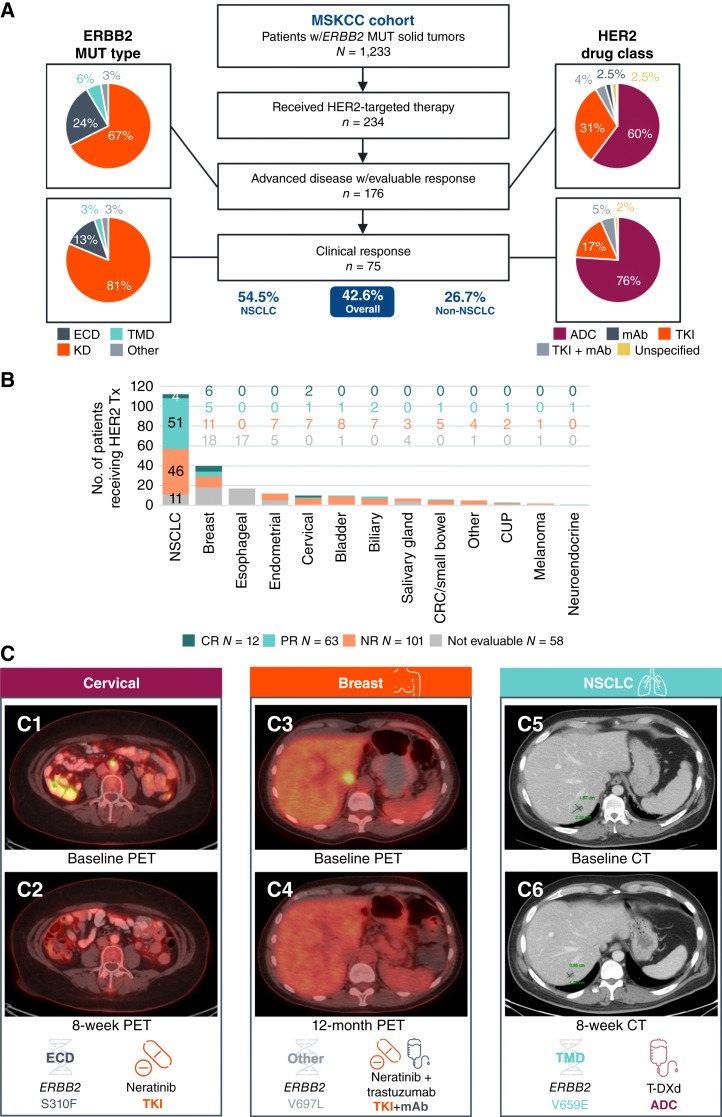
Clinical experience targeting *ERBB2* mutations. **A,** Flow diagram of MSKCC patients with *ERBB2* MUT tumors treated with HER2-directed therapies. The distributions of *ERBB2* mutation type and class of HER2-targeted therapy received are shown for response evaluable patients (*N* = 176) and for patients with clinical response (*N* = 75). “Other” *ERBB2* mutations in the clinical response group included an R678Q juxtamembrane domain mutation and a V697L mutation. **B,** Distribution of treated *ERBB2* MUT tumors across cancer types stratified by response. **C,** Representative tumor imaging from three patients with *ERBB2* MUT tumors. C1–C2: 59-year-old female with cervical cancer harboring *ERBB2* S310F mutation. PET scans of the retrocaval node performed at baseline and 8 weeks into treatment with neratinib are shown. C3–C4: 53-year-old female with metastatic breast cancer harboring *ERBB2* L755S mutation. PET scans of the liver at baseline and 12 months into treatment with neratinib plus trastuzumab are shown. C5–C6: 48-year-old male with lung cancer harboring *ERBB2* V659E mutation. CT scans of the liver at baseline and 8 weeks into trastuzumab emtansine (T-DM1) treatment are shown. ADC, antibody-drug conjugate; CR, complete response; CRC, colorectal cancer; CUP, cancer of unknown primary; ECD, extracellular domain; KD, kinase domain; mAB, monoclonal antibody; MUT, mutation (single-nucleotide variants and insertions/deletions); NSCLC, non-small cell lung cancer; NR, no response; PR, partial response; TKI, tyrosine kinase inhibitor; TMD, transmembrane domain.

We assessed the presence of activating *ERBB2* alterations across 83,332 solid tumors in the Memorial Sloan Kettering Cancer Center (MSKCC) clinicogenomic cohort (Supplementary Fig. S11A and S11B). *ERBB2* activating alterations were detected in 5.0% (*n* = 4,133) of tumors, and 234 of 1,233 (19.0%) patients with *ERBB2*mut solid tumors received one or more HER2-targeted therapies, including 17 patients with NSCLC enrolled on the DESTINY-Lung01 (NCT03505710) and DESTINY-Lung02 (NCT04644237) clinical trials who received T-DXd (Fig. 5A and B). Of the patients with advanced disease, 75.2% (176/234) were evaluable for clinical response. Clinical responses were observed in 42.6% (75/176) of patients with various cancer types which included 54.5% (55/101) of treated and evaluable patients with NSCLC and 26.7% (20/75) of treated and evaluable non-NSCLC patients. Responders harbored diverse *ERBB2* mutations, including mutations in the KD (81%, *n* = 61), ECD (13%, *n* = 10), TMD (3%, *n* = 2), and other regions (3%, *n* = 2). Seventy-six percent of responders received an ADC, 17% received a TKI, and 5% received combination TKI + mAb. Only 4 of 31 (12.9%) patients with a clinical response and available IHC results were HER2 IHC 3+, two of which (50.0%) were *ERBB2* co-amplified on NGS. Specifically, clinical responders to T-DXd (*n* = 36) included 28 patients with NSCLC with on-label *ERBB2*mut, 2 patients with NSCLC with off-label *ERBB2*mut (TMD V659E and an Ex20Ins (A775_G776insYLMA), both without HER2 IHC assessment and negative for *ERBB2* co-amplification by NGS), and 6 non-NSCLC patients (*N* = 2 with HER2 IHC 3+ and *N* = 4 that were HER2 IHC-negative/not assessed). Three representative patients from the MSKCC cohort experienced dramatic clinical and radiographic responses to various HER2-directed therapies, as discussed below (Fig. 5C).

#### Case 1

A 59-year-old female with metastatic cervical cancer was found to harbor an *ERBB2* S310F ECD mutation detected by tissue NGS. HER2 IHC was negative (1+) and *ERBB2* was non-amplified on NGS. Other notable genomic findings included a *PIK3CA* E545K mutation. Her cancer initially responded to 7 cycles of carboplatin, paclitaxel, and bevacizumab and progressed after an additional 11 cycles of bevacizumab maintenance therapy. The patient initiated the anti-HER2 TKI neratinib. Imaging after 8 weeks showed a complete metabolic response with a decrease in retrocaval nodal metastases by PET response criteria (Fig. 5, C1-C2). Her complete response continued before she ultimately progressed 17 months into neratinib treatment.

#### Case 2

A 53-year-old female with metastatic estrogen receptor–positive, HER2-negative (IHC 0) breast cancer was found to harbor an *ERBB2* V697L mutation identified on tissue NGS. *ERBB2* was non-amplified on NGS. After progression on first-line abemaciclib and letrozole, she initiated neratinib plus trastuzumab, achieving a complete metabolic response after 2 months of therapy. The complete response persisted for 73 months and is still ongoing (Fig. 5, C3-C4).

#### Case 3

A 48-year-old male neversmoker presented with stage IV, PD-L1–negative lung adenocarcinoma with metastatic disease to the liver, bone, and lungs. HER2 IHC status was not assessed. An *ERBB2* V659E TMD mutation—a mutation not included in the T-DXd CDx definition—was identified on tissue NGS, and *ERBB2* was not amplified. The patient started T-DXd after progression on neratinib. Follow-up imaging after 2 months of therapy demonstrated a partial response (−44%; Fig. 5, C5-C6) according to the RECIST guidelines (version 1.1). His partial response continued with further tumor shrinkage to −53% before progression after 7 months on treatment.

## Discussion

*ERBB2* (HER2) activation is an established oncogenic driver and key biomarker in multiple cancer types and is the biological basis of FDA approvals in breast cancer (trastuzumab, lapatinib, pertuzumab plus trastuzumab, trastuzumab emtansine, neratinib, T-DXd, tucatinib plus trastuzumab, and margetuximab), GEC (trastuzumab and T-DXd), NSCLC (T-DXd), CRC (tucatinib plus trastuzumab), and most recently pan-tumor (T-DXd; refs. 2–8). With the singular exception of T-DXd for *ERBB2*mut NSCLC, the biomarker associated with these approvals is HER2+ overexpression status assessed using IHC ± FISH. We demonstrate that the genomic mechanisms of *ERBB2* activation—inclusive of amplification, mutation, and rare intragenic rearrangements—vary across cancers and that this diverse spectrum of activating alteration types is detectable through CGP of both tissue and liquid biopsies.

*ERBB2* amplification was detected at relatively high frequencies in expected cancer types (e.g., GEC and breast), although these frequencies may diverge from expected frequencies of HER2+ tumors in these indications for various reasons, including (i) concordance between amplification calling on CGP and HER2 IHC/FISH and (ii) population biases in our cohort which tend toward advanced/metastatic disease. Although traditional methods of HER2 status assessment are still considered the gold standard, compelling studies suggest that assessment by CGP may offer additional benefit. In a study of advanced GEC, although the positive percent agreement of *ERBB2* AMP assessed by CGP compared with IHC ± FISH was low, concordant (HER2 IHC+/*ERBB2* AMP+) results were associated with significantly longer time to treatment discontinuation and overall survival than discordant (HER2 IHC+/*ERBB2* AMP−) results among patients treated with first-line trastuzumab (24). Moreover, multiple studies have demonstrated that the degree of *ERBB2* amplification as measured by quantitative copy number—a proxy for the degree of HER2 addiction in a tumor—is a predictor of response, with higher copy number associated with better outcomes (24–26). Thus, CGP may allow for more robust stratification of patients based on a more nuanced metric for HER2 overexpression/amplification status.

Whereas *ERBB2* activating mutations are already recognized as important biomarkers in NSCLC, we identified *ERBB2* mutations across solid tumor types with breast, CRC, bladder, and GEC cancers together accounting for 50.4% (4,301/8,526) of *ERBB2*mut tumors in our cohort. This represents a substantial population of patients who may also benefit from T-DXd and other HER2-targeted therapies. Together with NSCLC, these five cancer types comprised 69% (5,919/8,526) of *ERBB2*mut tumors. Even in the context of NSCLC, 26% of mutations reported as activating in our cohort—including TMD mutations, Ex16Alt, and a subset of Ex20Ins—would ostensibly be considered off-label according to the current T-DXd CDx for *ERBB2*mut NSCLC; we propose that these less common alterations warrant clinical investigation. Notably, 80.6% (4,768/5,919) of *ERBB2*mut tumors—including 84.7% (366/432) of NSCLC with T-DXd off-label *ERBB2* mutations—did not exhibit *ERBB2* co-amplification, suggesting that these may not be HER2 IHC 3+ and therefore would not be considered on-label for T-DXd according to the recent pan-tumor approval. Although 80% (1,252/1,565) of *ERBB2*mut breast cancer is non-amplified, the approval of T-DXd for hormone receptor–positive HER2-low/-ultralow cancers may modulate the added clinical value of identifying *ERBB2 *mutations in this cancer type context. In a small case series of breast tumors, we found that presence of an *ERBB2* mutation in the absence of amplification was associated with HER2-low IHC (IHC 1+ or IHC 2+/FISH−). This raises the question of whether *ERBB2* mutation may drive low levels of HER2 overexpression as a consequence of increased receptor cycling, as hypothesized previously (27).

Within the MSKCC clinicogenomic cohort, patients with diverse *ERBB2* activating mutations treated with various HER2 drug classes, including ADCs, experienced clinical responses in both NSCLC (54.5%) and non-NSCLC (26.7%) settings. The higher response rate in NSCLC may be explained by the greater percentage of patients who were treated with an ADC (84% of NSCLC vs. 35% of non-NSCLC) and with T-DXd, specifically (46% of NSCLC vs. 29% of non-NSCLC). Moreover, the NSCLC cohort included 17 patients treated with T-DXd on the DESTINYLung-01 and DESTINYLung-02 clinical trials and may therefore have had better performance characteristics than patients not enrolled on trials. Indeed, 13 of these 17 patients (76.5%) were clinical responders. Responders treated with T-DXd (*n* = 36) included two patients with NSCLC with off-label *ERBB2* mutations who were not assessed with IHC and four non-NSCLC patients who were HER2 IHC-negative/not assessed. Thus, 16.7% (6/36) of clinical responders to T-DXd would not have been identified without CGP. We also report case studies from the MSKCC clinical cohort demonstrating exceptional clinical responses to HER2-targeted therapies in a patient with NSCLC with a TMD mutation and in two patients with non-NSCLC and *ERBB2* mutations, all negative for *ERBB2* amplification/overexpression, as proof of principle that a wider lens should be used in determining eligibility for treatment with HER2-directed agents.

The landscape of *ERBB2* mutations varied substantially across cancer types at both the domain and codon levels. Meanwhile, there was less variation in the *ERBB3* mutation landscape with the majority of mutations localized to the ECD. This is expected given that HER3 is thought to possess an inactive KD, instead operating as an activator of other ERBB family members (especially HER2) through heterodimerization (28), an interaction which may be reliant on sites/structures found in the ECD (29). Importantly, liquid biopsy–based CGP was capable of detecting the spectrum of *ERBB2* activating alterations. Although sensitivity for amplifications was lower than for mutations, a known limitation of liquid biopsy due to variable ctDNA shed and/or sample heterogeneity which can yield noisy copy-number models and mask detection of low-level amplifications (17, 30), sensitivity was improved when the ctDNA content of samples was elevated (ctDNA TF ≥1% or ≥10%).

Given the size of our cohort, we were able to explore rare *ERBB2* activating alterations. Oncogenic TMD mutations (31) were detected in our cohort (0.08% prevalence pan-tumor), most commonly in NSCLC (59.4%, 98/165 TMD-mutated samples). Using an expanded case series, we confirmed prior observations concerning dual *ERBB2* and *EGFR*/*ERBB3* TMD mutations in NSCLC (32). Specifically, we noted that co-occurrence of *EGFR*/*ERBB3* TMD mutations seems to be allele-specific, with *ERBB2* TMD V > D and G > D changes frequently co-occurring (64.3%, 18/28), whereas V > E changes were mutually exclusive. These dual HER2/EGFR and HER2/HER3 TMD mutations are hypothesized to result in formation of a salt bridge, increasing heterodimerization, and may represent more aggressive activators than standalone *ERBB2* TMD mutations (32). We also report detection of genomic loss of *ERBB2* exon 16 (Ex16Alt) due to either intragenic deletion (Ex16Del) or splicing mutations (Ex16Splice) as a very rare (0.04% prevalence pan-tumor) but recurrent (33, 34) mechanism of *ERBB2* activation associated with resistance to targeted therapies in some cancers [e.g., osimertinib and other EGFR TKIs in NSCLC (35) and trastuzumab in GEC (36)]. Ex16Alt lead to expression of a HER2 isoform (33), HER2Δ16, expression of which has also been detected in the absence of underlying genomic events (34). HER2Δ16 lacks 16 amino acids in the juxtamembrane domain and has been proposed to cause an imbalance of cysteine residues in this region enabling homodimerization and constitutive activation of this HER2 isoform (37). We observed that Ex16Alt, particularly Ex16Del, frequently co-occurred with *ERBB2* amplification. If Ex16Alt represents the amplified allele, this could have implications for response to therapy. The predictive effects of these alterations regarding sensitivity/resistance to different HER2-targeted agents is unclear and deserving of clinical investigation. Although not analyzed in this study, reporting of other detected *ERBB2* rearrangements, e.g., intra/intergenic fusions, may also have predictive value. HER2 fusions have been reported in association with *ERBB2*-hyperamplified (high copy number) tumors and some may be oncogenic (38), whereas others may lack domains/sequences important for drug binding (38, 39).

Understanding the clinical implications of diverse *ERBB2* alteration types is complex. Efficacy of HER2-targeted therapy seems to be both tumor type– and alteration type–specific (as well as variant allele–specific within the subset of *ERBB2*mut tumors; ref. 10), and each therapy is associated with a unique spectrum of response/resistance (10, 40). Further confounding the predictive value of *ERBB2* alterations, various co-alterations, both primary and acquired, have been nominated as modulators of response to HER2-directed therapies. As an example of this multilayered complexity, a study of Chinese patients with breast cancer (which also included reanalysis of the MSK-BREAST, MutHER, and SUMMIT cohorts for verification) found that HER2-positive tumors with *TP53* co-mutation had reduced sensitivity to HER2 mAb regimens (trastuzumab, trastuzumab plus pertuzumab, and trastuzumab plus chemotherapy) but were sensitive to the TKI pyrotinib, whereas HER2-negative tumors with *ERBB2* mutation and *TP53* co-mutation were insensitive to pyrotinib (41, 42). Various reports have implicated *TP53* mutation [mAb vs. TKI in breast cancer (41, 42)], *PIK3CA* mutation [mAb in breast cancer (43); TKI in GEC (44)], *CCNE1* amplification [mAb and TKI in GEC (44, 45); mAb in breast cancer (46)], *RB1* loss [ADC in NSCLC (47)], *PTEN* loss [mAb in GEC (43, 48) and breast cancer (49)], *ERBB3* amplification/mutation [mAb, TKI, and ADC in breast cancer (50–53)], *EGFR* mutation [mAb and ADC in breast cancer (52, 53)], *MET* amplification/exon 14 mutation [mAb in breast cancer (54, 55) and TKI in GEC (44)], *IGF1R* amplification [mAb vs. TKI in breast cancer (56, 57)], and others as response-modifying alterations for HER2-targeted therapies based on both clinical and preclinical studies. However, the only genes with established resistance to a HER2-targeted therapy according to OncoKB (i.e., level R1 evidence) are *KRAS* and *NRAS*, which contraindicate treatment with tucatinib plus trastuzumab in the setting of metastatic CRC (19, 20). Given that resistance to HER2-targeted agents is heterogeneous and not fully understood, we conducted an exploratory analysis of previously reported response-modifying co-alterations with either *ERBB2* amplification or mutation(s) across select tumor types. A limitation of our study is that we are unable to distinguish between *de novo* and acquired resistance mutations due to the lack of clinical correlation. Whereas we report differences between co-alterations detected in *ERBB2*amp versus *ERBB2*mut tumors, it is important to consider that resistance variants may be more likely to arise in amplified cases as these are more frequently therapeutically targeted than mutations. In general, our study is limited by calling oncogenic alterations based on available evidence (see Supplementary Materials and Methods). Both functional studies and clinical correlation are necessary to definitively determine the clinical significance of *ERBB2* and *ERBB3* alterations (including rare mutation types, e.g., Ex16Alt and TMD MUT), as well as co-alterations, for response/resistance to HER2-directed therapies.

In summary, we present the diverse landscape of *ERBB2*/*ERBB3* activating alterations and co-alterations across >500,000 solid tumors which has implications for HER2/3-targeted therapies pan-tumor. *ERBB2* (HER2), with established significance as a biomarker in multiple tumor types and as the target of an extensive armamentarium of drugs, is exemplary of the critical need for contextualization in the field of precision medicine (58). Whereas singular biomarkers allow for enrichment for response in patient populations, accurate prediction of an individual’s response to a particular treatment will require an integrative approach which considers the pan-genomic (and ultimately multi-omic) profile of a tumor. Illustrating the benefit of such an approach, Randon and colleagues (59) demonstrated that more comprehensive negative selection of metastatic HER2+/*RAS*wt CRC (including identification of *ERBB2* co-alterations, other RTK/MAPK driver alterations, and/or *ERBB2* copy number <6 by NGS) for treatment with dual HER2 blockade (trastuzumab plus either pertuzumab or a TKI) stratified patients according to clinical outcomes. This may help to explain disappointing trials (60) in which a non-negligible proportion of HER2+/*RAS*wt patients did not derive clinical benefit from HER2-targeted agents. Although clear guidance on the predictive significance of each *ERBB2* alteration/coalteration/tumor type context is beyond the scope of this (and arguably any single) study, we sought to demonstrate that CGP provides more complete genomic information than the limited readout of HER2 ± overexpression/amplification provided by IHC/FISH. We propose that CGP is therefore critical for refining biomarker definitions, guiding clinical trial enrollment/treatment selection, and understanding variable response to HER2/3-targeted therapies pan-tumor. The low prevalence of *ERBB2* and *ERBB3* activating alterations across a variety of tumor types supports a tumor agnostic approach to investigations. Umbrella and/or basket trials using CGP could contribute to building a lexicon for organizing the currently byzantine HER2 therapy landscape.

## Supplementary Material

Supplementary Figure S1 Supplementary Figure S1. ERBB2/ERBB3 Amplification Pan-Tumor Landscape The distribution of amplification ratios for tumors with a) ERBB2 and b) ERBB3 amplification in tissue biopsies as assessed by FoundationOneCDx is shown. Amplification ratio is equal to the ratio of the modeled gene copy number to sample ploidy. Tumor types representing the highest proportion of ERBB2 or ERBB3 mutated tumors, respectively, which were the subject of focused analysis in this study are highlighted.

Supplementary Figure S2 Supplementary Figure S2. ERBB3 Activating Mutations In Select Cancers Distribution of ERBB3 mutations across key HER3 protein domains a) in the combined cohort of 5 major ERBB3 MUT cancer types and b) in each cancer type. The number of mutations is indicated. c) Lollipop plot mapping ERBB3 mutations across the HER3 protein in select cancer types. The two most frequent codon hotspots for each cancer type are labeled; % represent the proportion of observed mutations at a particular codon in the respective cancer type. AA, Amino Acid; ECD, Extracellular Domain; KD, Kinase Domain; MUT, Mutation (SNV, Indel); TMD, Transmembrane Domain.

Supplementary Figure S3 Supplementary Figure S3. ERBB2 Mutation Landscape Of Select Cancers Shown for each cancer type are (Left) the distribution of mutations across key HER2 protein domains, (Middle) lollipop plot showing the location and incidence of mutations across the HER2 protein (NM_004448) with the 5 most frequently mutated codons labeled, and (Right) plot showing distribution of mutations among the 10 most frequently mutated codons. The number of mutations is indicated. AA, Amino Acid; ECD, Extracellular Domain; KD, Kinase Domain; TMD, Transmembrane Domain.

Supplementary Figure S4 Supplementary Figure S4. ERBB3 Mutation Landscape Of Select Cancers Shown for each cancer type are (Left) the distribution of mutations across key HER3 protein domains, (Middle) lollipop plot showing the location and incidence of mutations across the HER3 protein (NM_001982) with the 5 most frequently mutated codons labeled, and (Right) plot showing distribution of mutations among the 10 most frequently mutated codons. The number of mutations is indicated. AA, Amino Acid; ECD, Extracellular Domain; KD, Kinase Domain; TMD, Transmembrane Domain.

Supplementary Figure S5 Supplementary Figure S5. Rare ERBB2 Activating Mutations In Select Cancers a) Distribution of ERBB2 TMD mutations combined across select cancers (Left) and number of TMD mutated tumors in select cancer types (Right). b) Distribution of Ex16Alt in the combined cohort (Left) and number of Ex16Alt tumors in select cancer types (Right). Ex16Alt, Exon 16 Alterations (Ex16 Deletion, Ex16 Splice Site); TMD, Transmembrane Domain.

Supplementary Figure S6 Supplementary Figure S6. ERBB2 Mutation Clonality Distribution of clonal fraction (VAF/TP) for ERBB2 mutation classes across select cancers. A dashed line at clonal fraction of 0.25 (25%) is shown as an estimated threshold for clonality1,2. ECD, Extracellular Domain; KD, Kinase Domain; TMD, Transmembrane Domain; TP, Computational Tumor Purity; VAF, Variant Allele Frequency. 1Sottoriva A, Graham TA. A pan-cancer signature of neutral tumor evolution. bioRxiv. 2015;014894. 2Bozic I, Gerold JM, Nowak MA. Quantifying Clonal and Subclonal Passenger Mutations in Cancer Evolution. PLoS Comput Biol. 2016;12:e1004731.

Supplementary Figure S7 Supplementary Figure S7. HER2 IHC Concordance In A Subset Of Breast Cancers With ERBB2-Activating Mutations (+) and (-) indicate the results of confirmatory FISH, if performed. ECD, Extracellular Domain; IDC, Invasive Ductal Carcinoma; ILC, Invasive Lobular Carcinoma; KD, Kinase Domain; NOS, Not Otherwise Specified

Supplementary Figure S8 Supplementary Figure S8. Co-Alteration Landscape Of ERBB2 Mutated Tumors In Select Cancers The prevalence of oncogenic co-alterations was compared for ERBB2 MUT (excluding samples w/ ERBB2 co-amplification) versus ERBB2 WT (non-mutated and non-amplified) a) NSCLC, b) Breast, c) CRC, d) Bladder, e) GEC, f) MSS CRC, and g) MSS GEC tumor samples profiled using tissue CGP. Only genes altered in at least 50 tumors and targeted across all tissue CGP assay versions were included. For each gene, mutations (SNVs, Indels), rearrangements, and copy number changes of known or likely functional significance detected using our assay were included. Genes highlighted in the National Comprehensive Cancer Network (NCCN) Guidelines as molecular drivers as well as genes altered at a high prevalence (≥10%) in either cohort are labeled for each volcano plot if a statistically significant difference was observed (p < 0.05, threshold indicated by dashed line). ERBB3, if statistically significant, is also labeled. Fisher’s Exact Test was performed to assess patterns of co-occurrence/mutual exclusivity between ERBB2 and other genes. P values were corrected using the Benjamini-Hochberg FDR method. MSS, Microsatellite Stable.

Supplementary Figure S9 Supplementary Figure S9. EGFR/ERBB2/ERBB3 TMD Mutation Co-Occurrence In NSCLC The inner circle represents ERBB2 TMD mutations detected in NSCLC tumors (n = 98). The outer circle represents co-occurring EGFR and ERBB3 TMD mutations detected in the same tumors. TMD, Transmembrane Domain.

Supplementary Figure S10 Supplementary Figure S10. HER2-Directed Therapy Response Modifying Alterations In Select Cancers (Related To Figure 4) Co-occurrence of alterations that have been reported to impact response to HER2-directed therapies (mAB, TKI, ADC) in tumors with ERBB2 amplification or ERBB2 activating mutations including TP53 MUT, PIK3CA MUT, CCNE1 AMP, RB1 MUT/DEL, PTEN MUT/DEL, ERBB3 AMP/MUT, EGFR MUT, MET AMP/Ex14 MUT, and IGF1R AMP. Patients with multiple ERBB2 alteration types (e.g., AMP + MUT) were excluded from the analysis. ADC, Antibody-Drug Conjugate; AMP, Amplification; mAB, Monoclonal Antibodies; MUT, Mutation (SNV, Indel); TKI, Tyrosine Kinase Inhibitor.

Supplementary Figure S11 Supplementary Figure S11. Pan-Tumor Landscape Of ERBB2 Activating Alterations Detected In The MSKCC Clinicogenomic Cohort a) Prevalence of ERBB2 activating alterations (known or likely functional significance) across solid tumors in the MSKCC clinicogenomic cohort. ‘Multiple’ includes patients with AMP + MUT, AMP + RE, MUT + RE, AMP + MUT + RE and patients with >1 MUT. b) Cancer type distribution of ERBB2 MUT tumors. AMP, Amplification (CN ≥ 6); MUT, Mutation (SNV, Indel); RE, Rearrangement.

Supplementary Table S1 Supplementary Table S1. Histology Breakdown Of Top 5 ERBB2 MUT Cancer Types

Supplementary Table S2 Supplementary Table S2. Histology Breakdown Of Top 5 ERBB3 MUT Cancer Types

Supplementary Data SUPPLEMENTARY MATERIALS & METHODS Foundation Medicine Comprehensive Genomic Profiling
