# Supplementary material for: Diverse ERBB2/ERBB3 Activating Alterations and Coalterations Have Implications for HER2/3-Targeted Therapies across Solid Tumors
Source: Cancer Res Commun. 2025 Apr 25;5(4):680–93. doi: 10.1158/2767-9764.CRC-24-0620 (PMC12022956; doi:10.1158/2767-9764.CRC-24-0620)
Supplement: Supplementary Figure S1 — ERBB2/ERBB3 Amplification Pan-Tumor Landscape The distribution of amplification ratios for tumors with a) ERBB2 and b) ERBB3 amplification in tissue biopsies as assessed by FoundationOneCDx is shown. Amplification ratio is equal to the ratio of the modeled gene copy number to sample ploidy. Tumor types representing the highest proportion of ERBB2 or ERBB3 mutated tumors, respectively, which were the subject of focused analysis in this study are highlighted. [file crc-24-0620_supplementary_figure_s1_suppsf1.pdf]

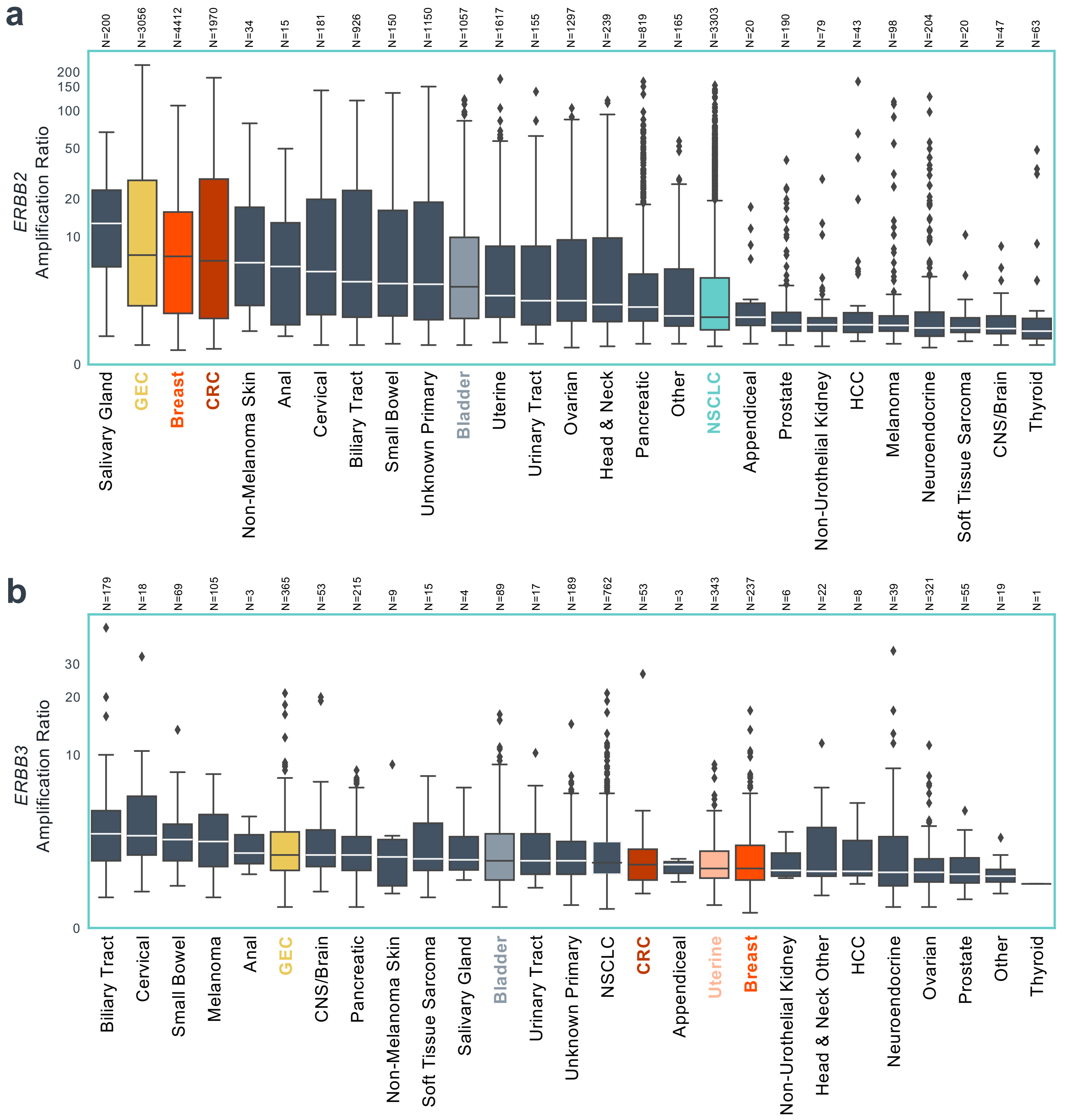

Supplementary Figure S1. *ERBB2*/*ERBB3* Amplification Pan-Tumor Landscape The distribution of amplification ratios for tumors with a) *ERBB2* and b) *ERBB3* amplification in tissue biopsies as assessed by FoundationOne®CDx is shown. Amplification ratio is equal to the ratio of the modeled gene copy number to sample ploidy. Tumor types representing the highest proportion of *ERBB2* or *ERBB3* mutated tumors, respectively, which were the subject of focused analysis in this study are highlighted.
