# Supplementary material for: Diverse ERBB2/ERBB3 Activating Alterations and Coalterations Have Implications for HER2/3-Targeted Therapies across Solid Tumors
Source: Cancer Res Commun. 2025 Apr 25;5(4):680–93. doi: 10.1158/2767-9764.CRC-24-0620 (PMC12022956; doi:10.1158/2767-9764.CRC-24-0620)
Supplement: Supplementary Figure S2 — ERBB3 Activating Mutations In Select Cancers Distribution of ERBB3 mutations across key HER3 protein domains a) in the combined cohort of 5 major ERBB3 MUT cancer types and b) in each cancer type. The number of mutations is indicated. c) Lollipop plot mapping ERBB3 mutations across the HER3 protein in select cancer types. The two most frequent codon hotspots for each cancer type are labeled; % represent the proportion of observed mutations at a particular codon in the respective cancer type. AA, Amino Acid; ECD, Extracellular Domain; KD, Kinase Domain; MUT, Mutation (SNV, Indel); TMD, Transmembrane Domain. [file crc-24-0620_supplementary_figure_s2_suppsf2.pdf]

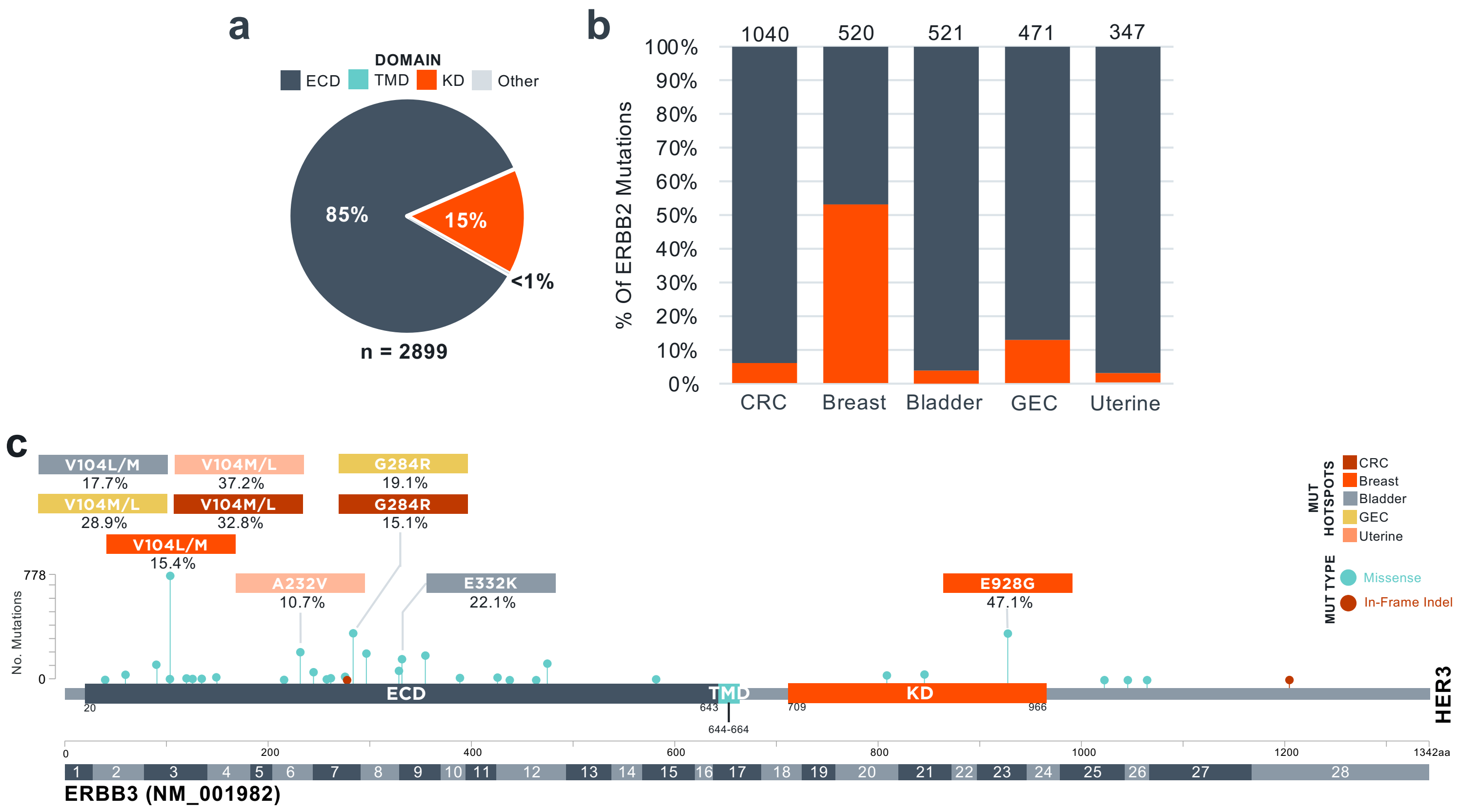

Supplementary Figure S2. *ERBB3* Activating Mutations In Select Cancers Distribution of *ERBB3* mutations across key HER3 protein domains a) in the combined cohort of 5 major *ERBB3* MUT cancer types and b) in each cancer type. The number of mutations is indicated. c) Lollipop plot mapping *ERBB3* mutations across the HER3 protein in select cancer types. The two most frequent codon hotspots for each cancer type are labeled; % represent the proportion of observed mutations at a particular codon in the respective cancer type. AA, Amino Acid; ECD, Extracellular Domain; KD, Kinase Domain; MUT, Mutation (SNV, Indel); TMD, Transmembrane Domain.
