# Supplementary material for: Diverse ERBB2/ERBB3 Activating Alterations and Coalterations Have Implications for HER2/3-Targeted Therapies across Solid Tumors
Source: Cancer Res Commun. 2025 Apr 25;5(4):680–93. doi: 10.1158/2767-9764.CRC-24-0620 (PMC12022956; doi:10.1158/2767-9764.CRC-24-0620)
Supplement: Supplementary Figure S3 — ERBB2 Mutation Landscape Of Select Cancers Shown for each cancer type are (Left) the distribution of mutations across key HER2 protein domains, (Middle) lollipop plot showing the location and incidence of mutations across the HER2 protein (NM_004448) with the 5 most frequently mutated codons labeled, and (Right) plot showing distribution of mutations among the 10 most frequently mutated codons. The number of mutations is indicated. AA, Amino Acid; ECD, Extracellular Domain; KD, Kinase Domain; TMD, Transmembrane Domain. [file crc-24-0620_supplementary_figure_s3_suppsf3.pdf]

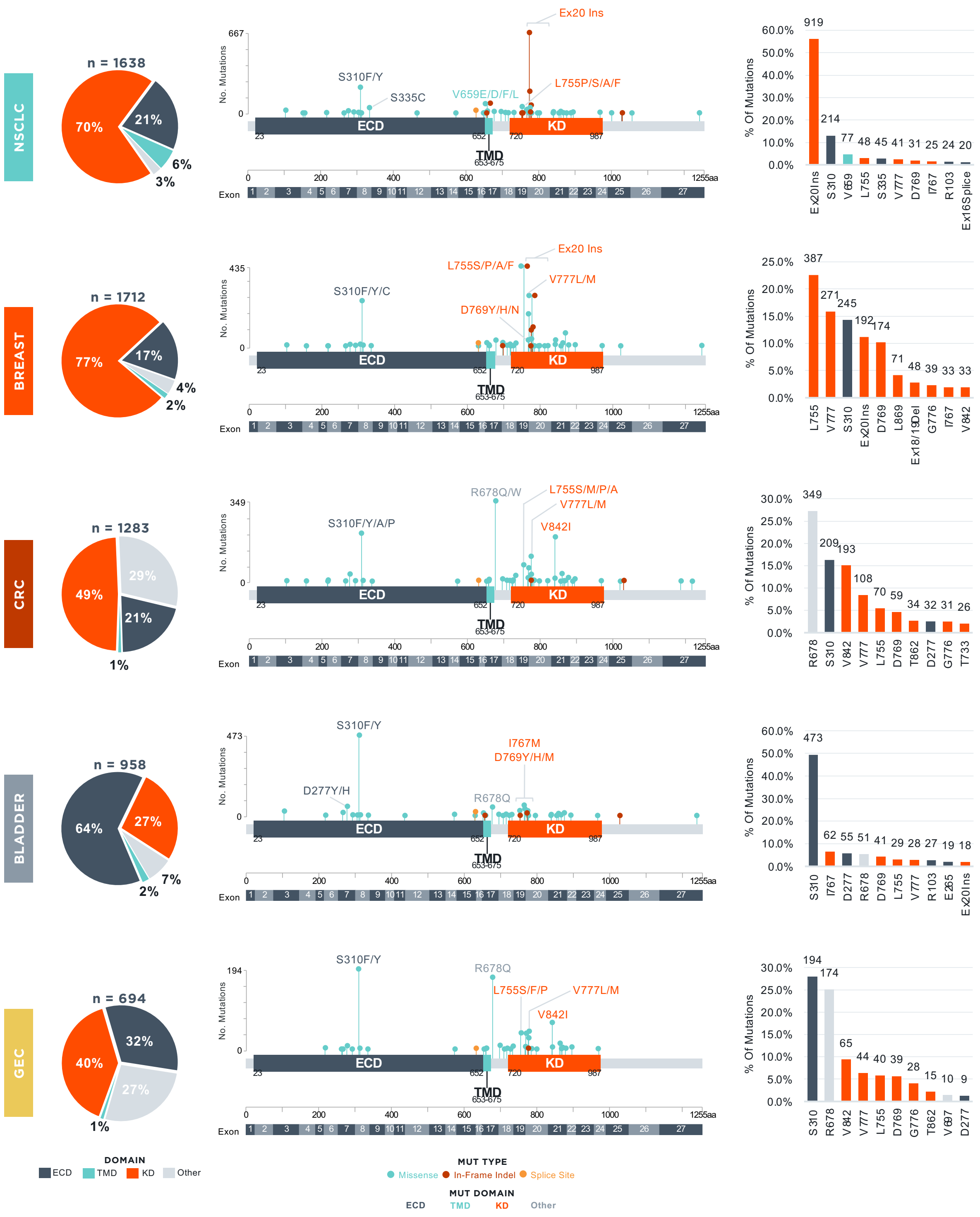

Supplementary Figure S3. ***ERBB2*** **Mutation Landscape Of Select Cancers** Shown for each cancer type are (Left) the distribution of mutations across key HER2 protein domains, (Middle) lollipop plot showing the location and incidence of mutations across the HER2 protein (NM_004448) with the 5 most frequently mutated codons labeled, and (Right) plot showing distribution of mutations among the 10 most frequently mutated codons. The number of mutations is indicated. AA, Amino Acid; ECD, Extracellular Domain; KD, Kinase Domain; TMD, Transmembrane Domain.
