# Supplementary material for: Diverse ERBB2/ERBB3 Activating Alterations and Coalterations Have Implications for HER2/3-Targeted Therapies across Solid Tumors
Source: Cancer Res Commun. 2025 Apr 25;5(4):680–93. doi: 10.1158/2767-9764.CRC-24-0620 (PMC12022956; doi:10.1158/2767-9764.CRC-24-0620)
Supplement: Supplementary Figure S5 — Rare ERBB2 Activating Mutations In Select Cancers a) Distribution of ERBB2 TMD mutations combined across select cancers (Left) and number of TMD mutated tumors in select cancer types (Right). b) Distribution of Ex16Alt in the combined cohort (Left) and number of Ex16Alt tumors in select cancer types (Right). Ex16Alt, Exon 16 Alterations (Ex16 Deletion, Ex16 Splice Site); TMD, Transmembrane Domain. [file crc-24-0620_supplementary_figure_s5_suppsf5.pdf]

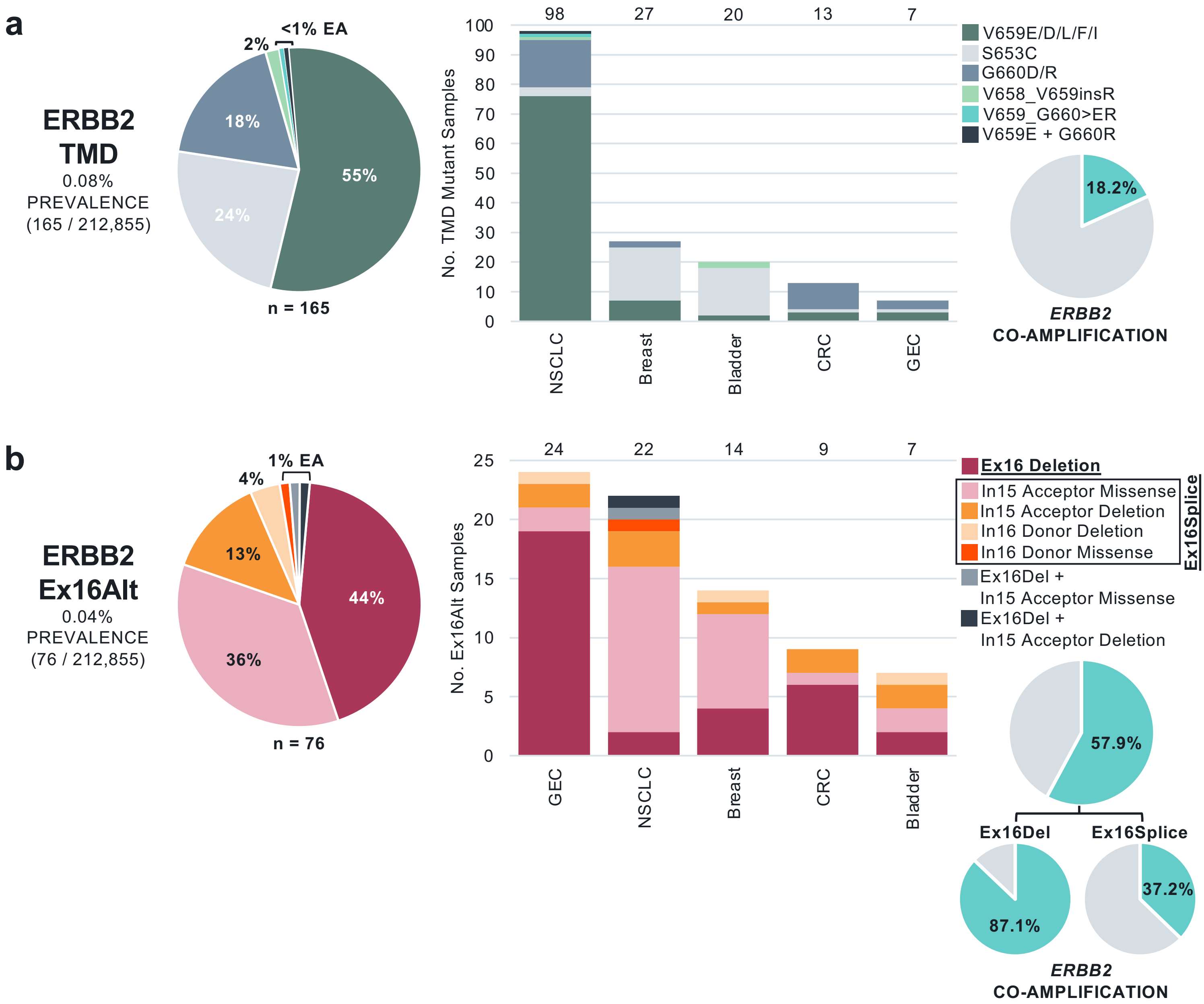

Supplementary Figure S5. Rare *ERBB2* Activating Mutations In Select Cancers a) Distribution of *ERBB2* TMD mutations combined across select cancers (Left) and number of TMD mutated tumors in select cancer types (Right). b) Distribution of Ex16Alt in the combined cohort (Left) and number of Ex16Alt tumors in select cancer types (Right). Ex16Alt, Exon 16 Alterations (Ex16 Deletion, Ex16 Splice Site); TMD, Transmembrane Domain.
