# Supplementary material for: Diverse ERBB2/ERBB3 Activating Alterations and Coalterations Have Implications for HER2/3-Targeted Therapies across Solid Tumors
Source: Cancer Res Commun. 2025 Apr 25;5(4):680–93. doi: 10.1158/2767-9764.CRC-24-0620 (PMC12022956; doi:10.1158/2767-9764.CRC-24-0620)
Supplement: Supplementary Figure S6 — ERBB2 Mutation Clonality Distribution of clonal fraction (VAF/TP) for ERBB2 mutation classes across select cancers. A dashed line at clonal fraction of 0.25 (25%) is shown as an estimated threshold for clonality1,2. ECD, Extracellular Domain; KD, Kinase Domain; TMD, Transmembrane Domain; TP, Computational Tumor Purity; VAF, Variant Allele Frequency. 1Sottoriva A, Graham TA. A pan-cancer signature of neutral tumor evolution. bioRxiv. 2015;014894. 2Bozic I, Gerold JM, Nowak MA. Quantifying Clonal and Subclonal Passenger Mutations in Cancer Evolution. PLoS Comput Biol. 2016;12:e1004731. [file crc-24-0620_supplementary_figure_s6_suppsf6.pdf]

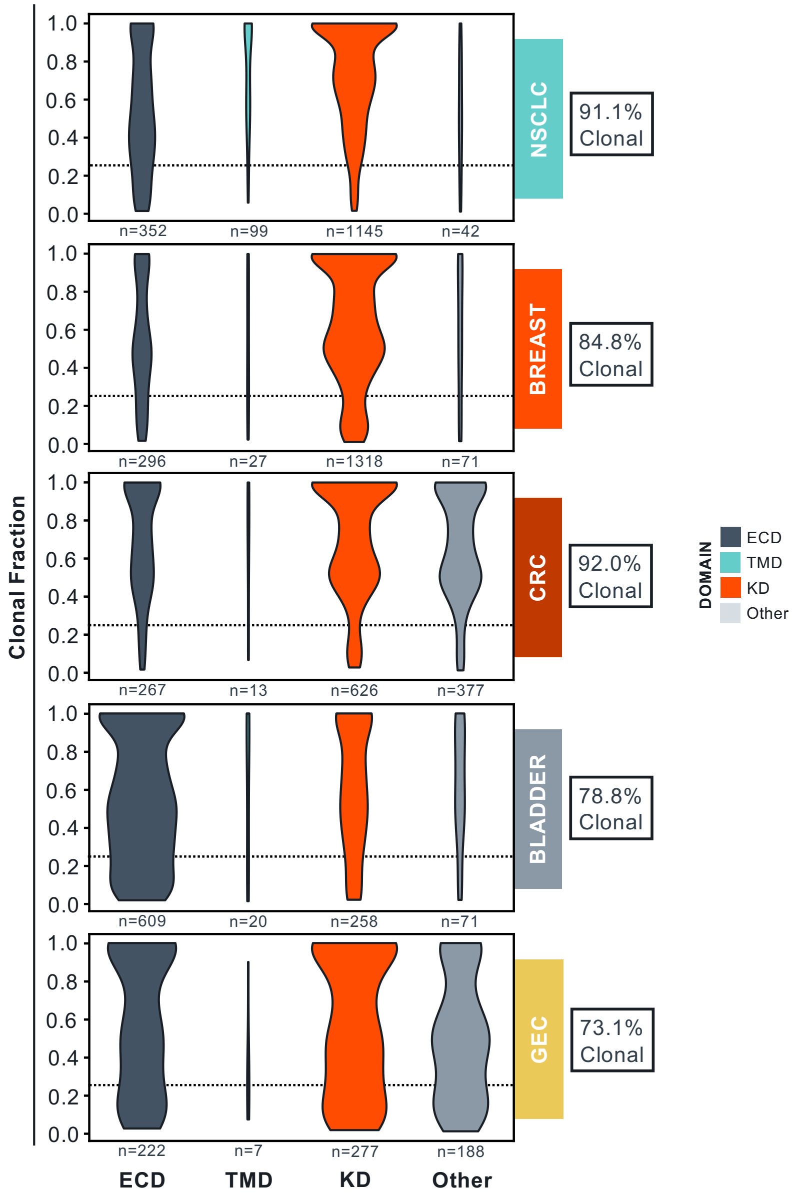


**Supplementary Figure S6. *ERBB2* Mutation Clonality** Distribution of clonal fraction (VAF/TP) for *ERBB2* mutation classes across select cancers. A dashed line at clonal fraction of 0.25 (25%) is shown as an estimated threshold for clonality[1,2]. ECD, Extracellular Domain; KD, Kinase Domain; TMD, Transmembrane Domain; TP, Computational Tumor Purity; VAF, Variant Allele Frequency. [1]Sottoriva A, Graham TA. A pan-cancer signature of neutral tumor evolution. bioRxiv. 2015;014894. [2]Bozic I, Gerold JM, Nowak MA. Quantifying Clonal and Subclonal Passenger Mutations in Cancer Evolution. PLoS Comput Biol. 2016;12:e1004731.
