# Supplementary material for: Diverse ERBB2/ERBB3 Activating Alterations and Coalterations Have Implications for HER2/3-Targeted Therapies across Solid Tumors
Source: Cancer Res Commun. 2025 Apr 25;5(4):680–93. doi: 10.1158/2767-9764.CRC-24-0620 (PMC12022956; doi:10.1158/2767-9764.CRC-24-0620)
Supplement: Supplementary Figure S7 — HER2 IHC Concordance In A Subset Of Breast Cancers With ERBB2-Activating Mutations (+) and (-) indicate the results of confirmatory FISH, if performed. ECD, Extracellular Domain; IDC, Invasive Ductal Carcinoma; ILC, Invasive Lobular Carcinoma; KD, Kinase Domain; NOS, Not Otherwise Specified [file crc-24-0620_supplementary_figure_s7_suppsf7.pdf]

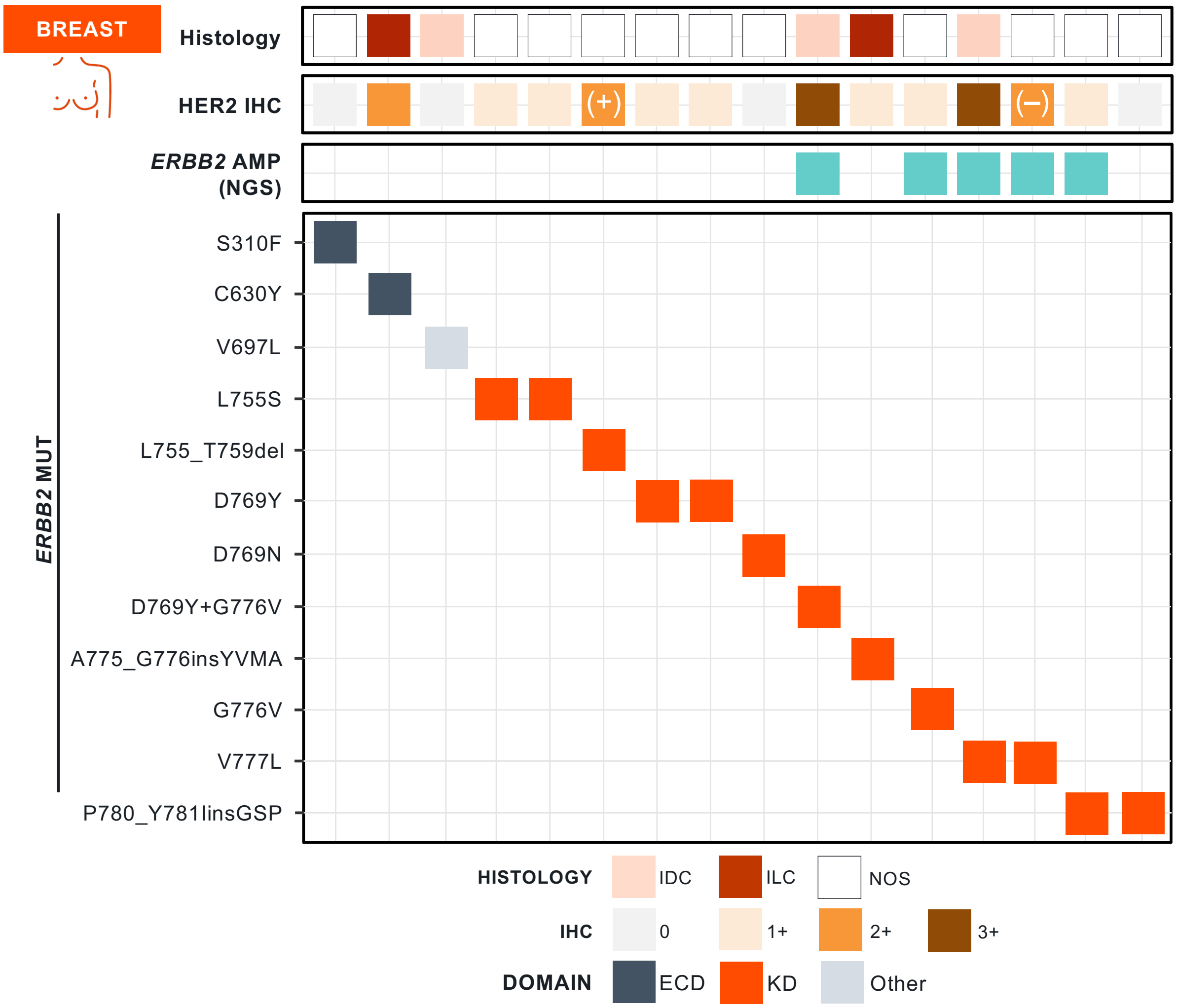

Supplementary Figure S7. HER2 IHC Concordance In A Subset Of Breast Cancers With *ERBB2*-Activating Mutations (+) and (-) indicate the results of confirmatory FISH, if performed. ECD, Extracellular Domain; IDC, Invasive Ductal Carcinoma; ILC, Invasive Lobular Carcinoma; KD, Kinase Domain; NOS, Not Otherwise Specified
