# Supplementary material for: Diverse ERBB2/ERBB3 Activating Alterations and Coalterations Have Implications for HER2/3-Targeted Therapies across Solid Tumors
Source: Cancer Res Commun. 2025 Apr 25;5(4):680–93. doi: 10.1158/2767-9764.CRC-24-0620 (PMC12022956; doi:10.1158/2767-9764.CRC-24-0620)
Supplement: Supplementary Figure S8 — Co-Alteration Landscape Of ERBB2 Mutated Tumors In Select Cancers The prevalence of oncogenic co-alterations was compared for ERBB2 MUT (excluding samples w/ ERBB2 co-amplification) versus ERBB2 WT (non-mutated and non-amplified) a) NSCLC, b) Breast, c) CRC, d) Bladder, e) GEC, f) MSS CRC, and g) MSS GEC tumor samples profiled using tissue CGP. Only genes altered in at least 50 tumors and targeted across all tissue CGP assay versions were included. For each gene, mutations (SNVs, Indels), rearrangements, and copy number changes of known or likely functional significance detected using our assay were included. Genes highlighted in the National Comprehensive Cancer Network (NCCN) Guidelines as molecular drivers as well as genes altered at a high prevalence (≥10%) in either cohort are labeled for each volcano plot if a statistically significant difference was observed (p < 0.05, threshold indicated by dashed line). ERBB3, if statistically significant, is also labeled. Fisher’s Exact Test was performed to assess patterns of co-occurrence/mutual exclusivity between ERBB2 and other genes. P values were corrected using the Benjamini-Hochberg FDR method. MSS, Microsatellite Stable. [file crc-24-0620_supplementary_figure_s8_suppsf8.pdf]

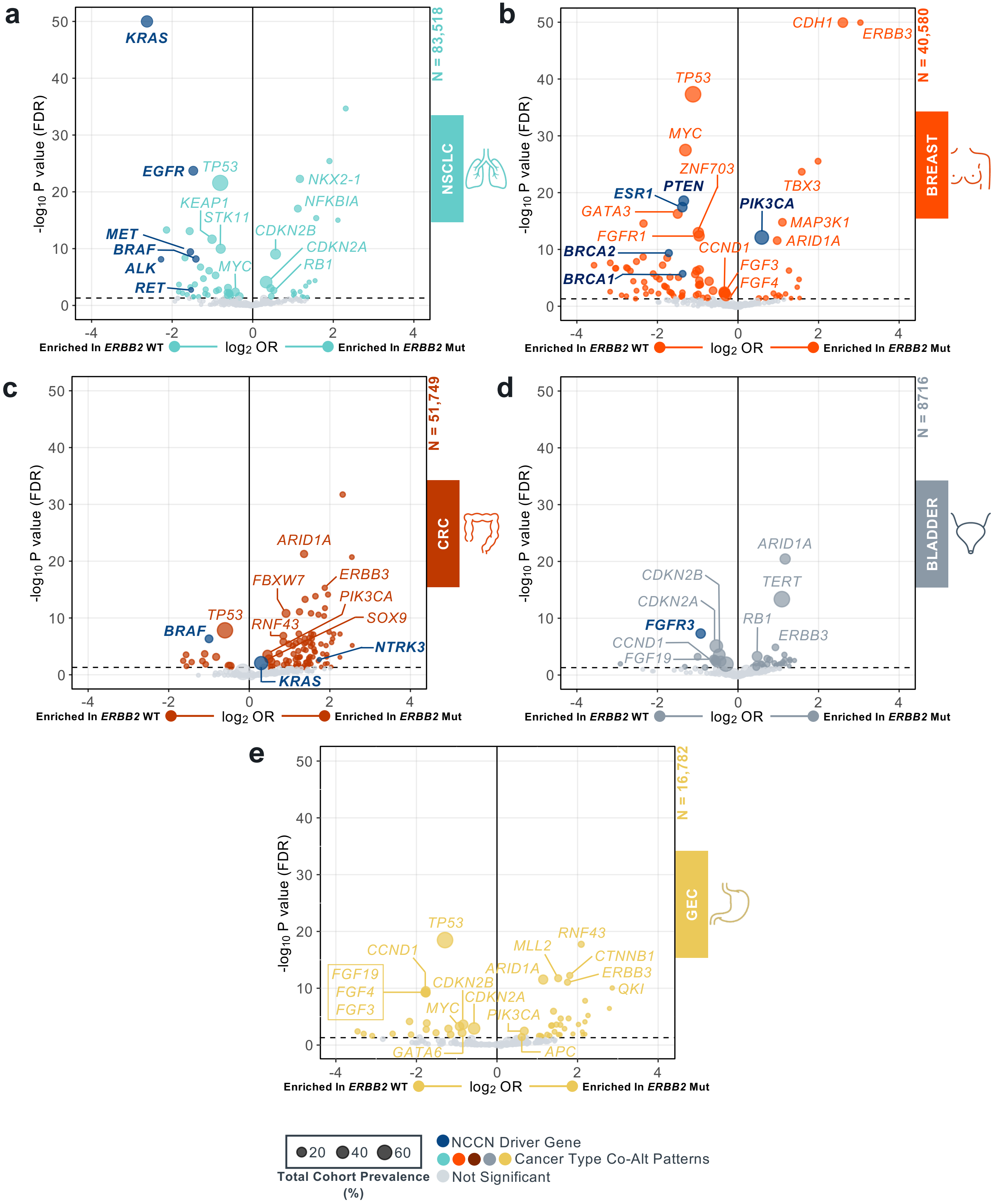
a
N = 83,518
NSCLC
KRAS
EGFR
TP53
NKX2-1
NFKBIA
KEAP1
STK11
MET
BRAF
ALK
RET
MYC
CDKN2B
CDKN2A
RB1
-log10 P value (FDR)
Enriched In ERBB2 WT
log2 OR
Enriched In ERBB2 Mut
b
N = 40,580
BREAST
CDH1
ERBB3
TP53
MYC
ZNF703
PTEN
ESR1
GATA3
TBX3
PIK3CA
MAP3K1
ARID1A
FGFR1
BRCA2
CCND1
BRCA1
FGF3
FGF4
c
N = 51,749
CRC
ARID1A
ERBB3
FBXW7
PIK3CA
TP53
SOX9
RNF43
BRAF
NTRK3
KRAS
d
N = 8716
BLADDER
ARID1A
CDKN2B
TERT
CDKN2A
RB1
FGFR3
ERBB3
CCND1
FGF19
e
N = 16,782
GEC
TP53
RNF43
CCND1
MLL2
CTNNB1
ARID1A
ERBB3
FGF19
FGF4
FGF3
CDKN2B
QKI
CDKN2A
MYC
PIK3CA
GATA6
APC
20
40
60
Total Cohort Prevalence (%)
NCCN Driver Gene
Cancer Type Co-Alt Patterns
Not Significant

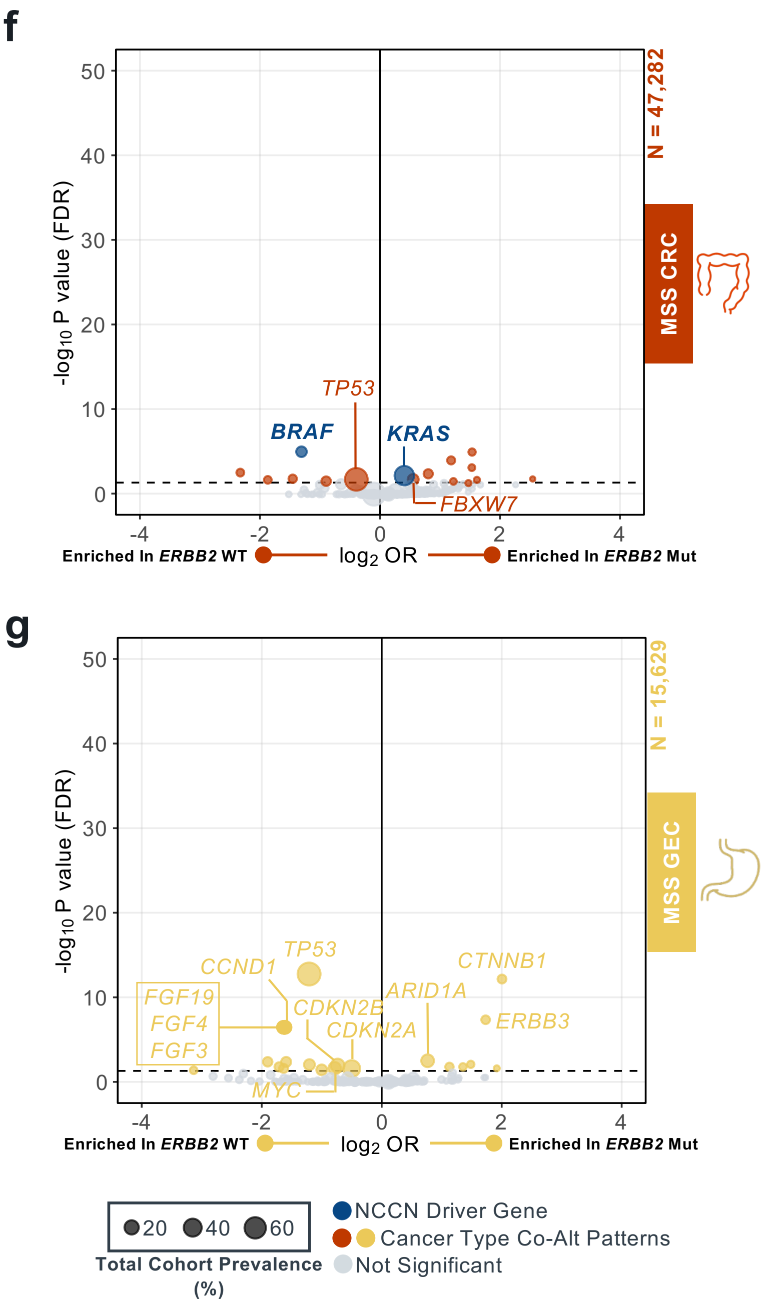

Supplementary Figure S8. Co-Alteration Landscape Of *ERBB2* Mutated Tumors In Select Cancers The prevalence of oncogenic co-alterations was compared for *ERBB2* MUT (excluding samples w/ *ERBB2* co-amplification) versus *ERBB2* WT (non-mutated and non-amplified) a) NSCLC, b) Breast, c) CRC, d) Bladder, e) GEC, f) MSS CRC, and g) MSS GEC tumor samples profiled using tissue CGP. Only genes altered in at least 50 tumors and targeted across all tissue CGP assay versions were included. For each gene, mutations (SNVs, Indels), rearrangements, and copy number changes of known or likely functional significance detected using our assay were included. Genes highlighted in the National Comprehensive Cancer Network (NCCN) Guidelines as molecular drivers as well as genes altered at a high prevalence (≥10%) in either cohort are labeled for each volcano plot if a statistically significant difference was observed ($p < 0.05$, threshold indicated by dashed line). *ERBB3*, if statistically significant, is also labeled. Fisher's Exact Test was performed to assess patterns of co-occurrence/mutual exclusivity between *ERBB2* and other genes. P values were corrected using the Benjamini-Hochberg FDR method. MSS, Microsatellite Stable.
