# Supplementary material for: Diverse ERBB2/ERBB3 Activating Alterations and Coalterations Have Implications for HER2/3-Targeted Therapies across Solid Tumors
Source: Cancer Res Commun. 2025 Apr 25;5(4):680–93. doi: 10.1158/2767-9764.CRC-24-0620 (PMC12022956; doi:10.1158/2767-9764.CRC-24-0620)
Supplement: Supplementary Figure S9 — EGFR/ERBB2/ERBB3 TMD Mutation Co-Occurrence In NSCLC The inner circle represents ERBB2 TMD mutations detected in NSCLC tumors (n = 98). The outer circle represents co-occurring EGFR and ERBB3 TMD mutations detected in the same tumors. TMD, Transmembrane Domain. [file crc-24-0620_supplementary_figure_s9_suppsf9.pdf]

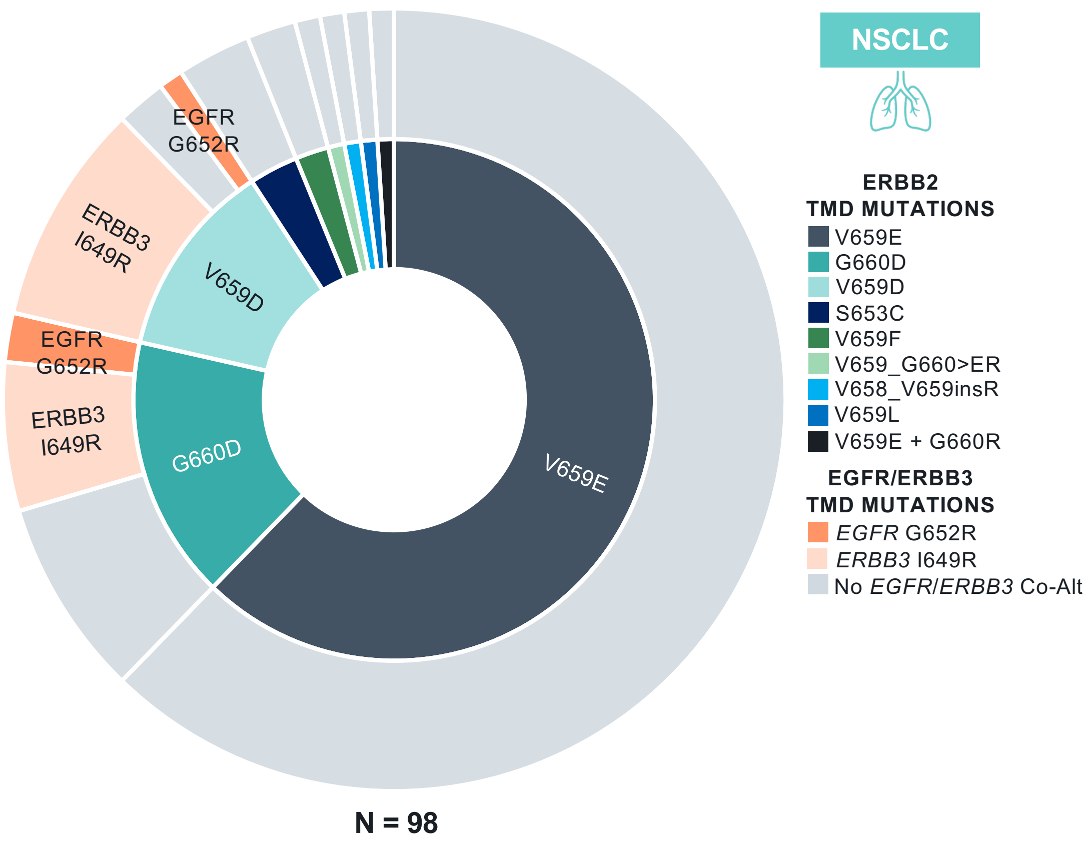

Supplementary Figure S9. *EGFR/ERBB2/ERBB3* TMD Mutation Co-Occurrence In NSCLC The inner circle represents *ERBB2* TMD mutations detected in NSCLC tumors (n = 98). The outer circle represents co-occurring *EGFR* and *ERBB3* TMD mutations detected in the same tumors. TMD, Transmembrane Domain.
