# Supplementary material for: Diverse ERBB2/ERBB3 Activating Alterations and Coalterations Have Implications for HER2/3-Targeted Therapies across Solid Tumors
Source: Cancer Res Commun. 2025 Apr 25;5(4):680–93. doi: 10.1158/2767-9764.CRC-24-0620 (PMC12022956; doi:10.1158/2767-9764.CRC-24-0620)
Supplement: Supplementary Figure S10 — HER2-Directed Therapy Response Modifying Alterations In Select Cancers (Related To Figure 4) Co-occurrence of alterations that have been reported to impact response to HER2-directed therapies (mAB, TKI, ADC) in tumors with ERBB2 amplification or ERBB2 activating mutations including TP53 MUT, PIK3CA MUT, CCNE1 AMP, RB1 MUT/DEL, PTEN MUT/DEL, ERBB3 AMP/MUT, EGFR MUT, MET AMP/Ex14 MUT, and IGF1R AMP. Patients with multiple ERBB2 alteration types (e.g., AMP + MUT) were excluded from the analysis. ADC, Antibody-Drug Conjugate; AMP, Amplification; mAB, Monoclonal Antibodies; MUT, Mutation (SNV, Indel); TKI, Tyrosine Kinase Inhibitor. [file crc-24-0620_supplementary_figure_s10_suppsf10.pdf]

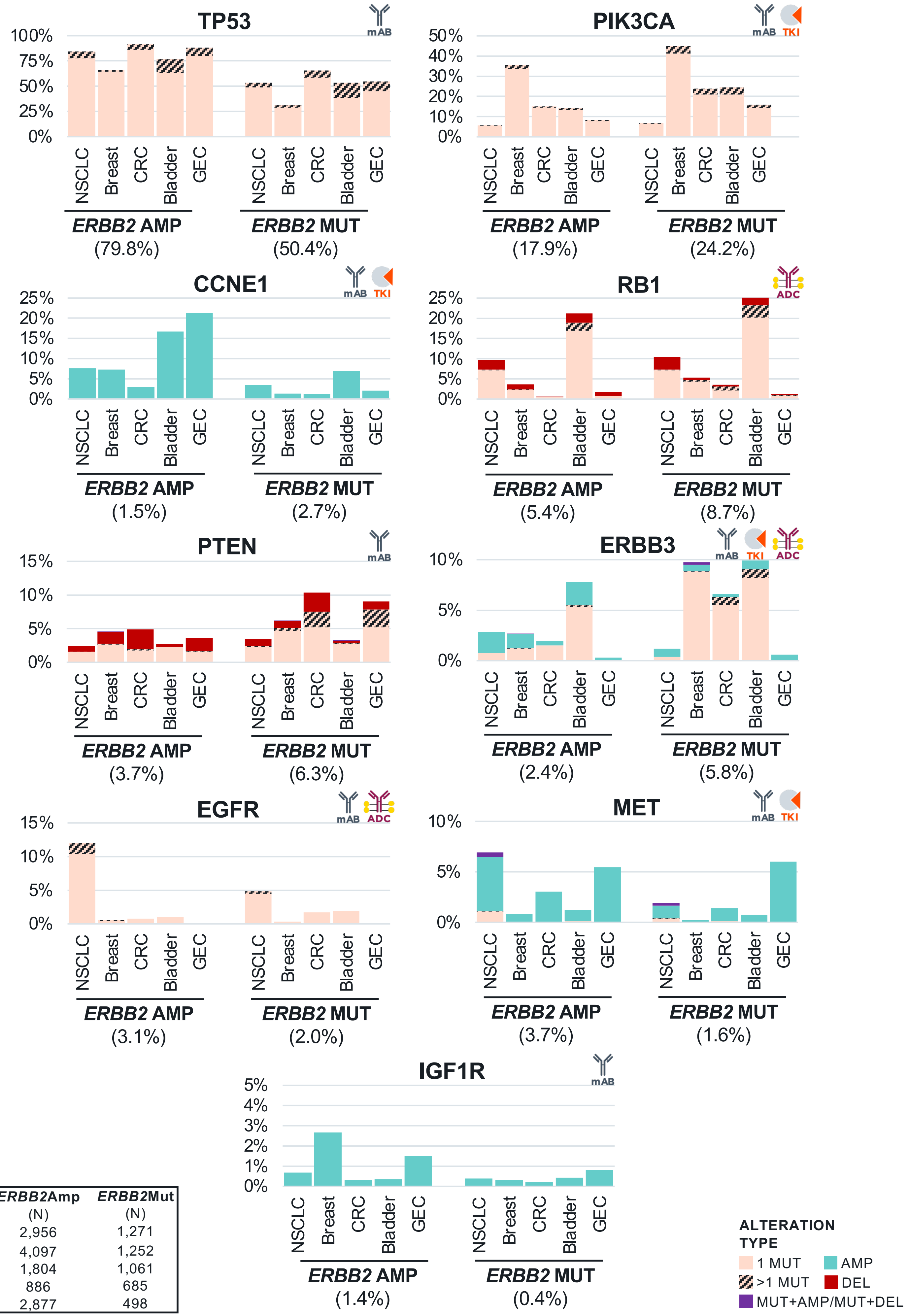

	*ERBB2*Amp (N)	*ERBB2*Mut (N)
**NSCLC**	2,956	1,271
**Breast**	4,097	1,252
**CRC**	1,804	1,061
**Bladder**	886	685
**GEC**	2,877	498

Supplementary Figure S10. HER2-Directed Therapy Response Modifying Alterations In Select Cancers (Related To Figure 4) Co-occurrence of alterations that have been reported to impact response to HER2-directed therapies (mAB, TKI, ADC) in tumors with *ERBB2* amplification or *ERBB2* activating mutations including *TP53* MUT, *PIK3CA* MUT, *CCNE1* AMP, *RB1* MUT/DEL, *PTEN* MUT/DEL, *ERBB3* AMP/MUT, *EGFR* MUT, *MET* AMP/Ex14 MUT, and *IGF1R* AMP. Patients with multiple *ERBB2* alteration types (e.g., AMP + MUT) were excluded from the analysis. ADC, Antibody-Drug Conjugate; AMP, Amplification; mAB, Monoclonal Antibodies; MUT, Mutation (SNV, Indel); TKI, Tyrosine Kinase Inhibitor.

	TP53	***PIK3CA***	***CCNE1***	***RB1***	***PTEN***	***ERBB3***	***EGFR***	***MET***	***IGF1R***
**REFERENCES**	(41) Liu et al. 2022; (42) Fedorova et al. 2020	(44) Kim et al. 2014; (43) Berns et al. 2007	(45) Lee et al. 2015; (44) Kim et al. 2014; (46) Scaltriti et al. 2011	(47) Gupta et al. 2023	(49) Yokoyama et al. 2021; (43) Berns et al. 2007; (48) Nagata et al. 2004	(51) Hanker et al. 2021; (50) Smyth et al. 2020; (52) Schwarz et al. 2017; (53) Ritter et al. 2007	(52) Schwarz et al. 2017; (53) Ritter et al. 2007	(44) Kim et al 2014; (55) Paulson et al. 2013; (54) Shattuck et al. 2008	(57) Nahta et al 2007; (56) Nahta et al. 2005
