# Supplementary material for: Diverse ERBB2/ERBB3 Activating Alterations and Coalterations Have Implications for HER2/3-Targeted Therapies across Solid Tumors
Source: Cancer Res Commun. 2025 Apr 25;5(4):680–93. doi: 10.1158/2767-9764.CRC-24-0620 (PMC12022956; doi:10.1158/2767-9764.CRC-24-0620)
Supplement: Supplementary Figure S11 — Pan-Tumor Landscape Of ERBB2 Activating Alterations Detected In The MSKCC Clinicogenomic Cohort a) Prevalence of ERBB2 activating alterations (known or likely functional significance) across solid tumors in the MSKCC clinicogenomic cohort. ‘Multiple’ includes patients with AMP + MUT, AMP + RE, MUT + RE, AMP + MUT + RE and patients with >1 MUT. b) Cancer type distribution of ERBB2 MUT tumors. AMP, Amplification (CN ≥ 6); MUT, Mutation (SNV, Indel); RE, Rearrangement. [file crc-24-0620_supplementary_figure_s11_suppsf11.pdf]

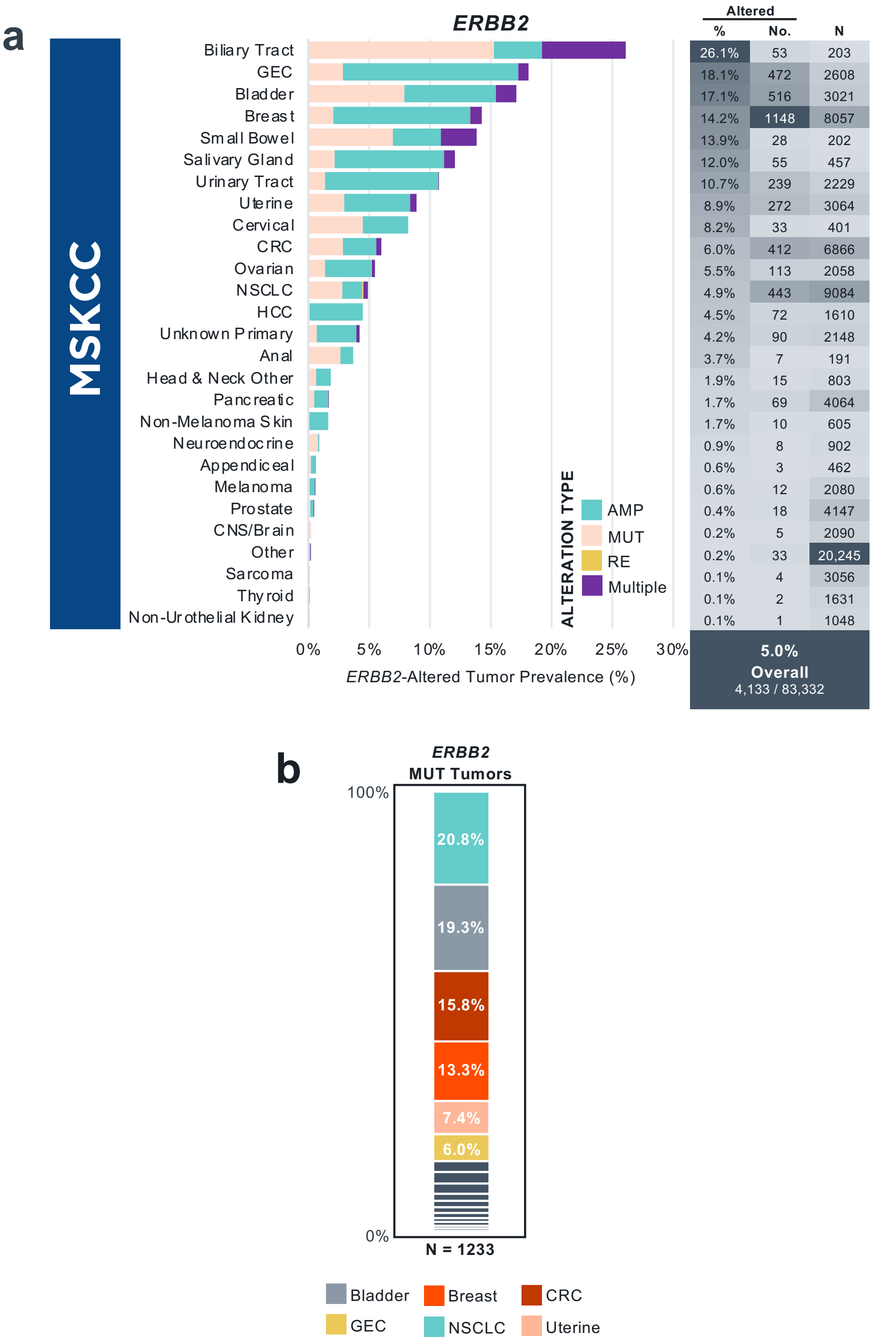

Supplementary Figure S11. Pan-Tumor Landscape Of *ERBB2* Activating Alterations Detected In The MSKCC Clinicogenomic Cohort a) Prevalence of *ERBB2* activating alterations (known or likely functional significance) across solid tumors in the MSKCC clinicogenomic cohort. 'Multiple' includes patients with AMP + MUT, AMP + RE, MUT + RE, AMP + MUT + RE and patients with >1 MUT. b) Cancer type distribution of *ERBB2* MUT tumors. AMP, Amplification (CN ≥ 6); MUT, Mutation (SNV, Indel); RE, Rearrangement.
