# Supplementary material for: Diverse ERBB2/ERBB3 Activating Alterations and Coalterations Have Implications for HER2/3-Targeted Therapies across Solid Tumors
Source: Cancer Res Commun. 2025 Apr 25;5(4):680–93. doi: 10.1158/2767-9764.CRC-24-0620 (PMC12022956; doi:10.1158/2767-9764.CRC-24-0620)
Supplement: Supplementary Table S1 — Histology Breakdown Of Top 5 ERBB2 MUT Cancer Types [file crc-24-0620_supplementary_table_s1_suppst1.pdf]

Supplementary Table S1. Histology Breakdown Of Top 5 *ERBB2* MUT Cancer Types

HISTOLOGY	ERBB2 ALT PREVALENCE	% ERBB2 ALT AMP	MUT/RE	MULTIPLE	N
**NSCLC**	**5.3%**	**64.6%**	**27.8%**	**7.6%**	**N=85,602**
lung adenocarcinoma	6.4%	59.6%	31.9%	8.6%	53,636
lung squamous cell carcinoma (SCC)	5.5%	50.0%	32.4%	17.6%	615
lung non-small cell lung carcinoma, NOS	4.7%	77.5%	16.8%	5.7%	12,511
lung large cell neuroendocrine carcinoma	3.9%	81.8%	9.1%	9.1%	280
lung adenosquamous carcinoma	3.6%	50.0%	50.0%	0.0%	56
lung sarcomatoid carcinoma	2.9%	83.3%	13.8%	2.8%	16,710
lung large cell carcinoma	2.3%	83.3%	16.7%	0.0%	527
lung carcinosarcoma	2.1%	88.5%	7.7%	3.8%	1,265
lung lymphoepithelioma	0.0%	-	-	-	2
**Breast**	**12.7%**	**72.3%**	**22.1%**	**5.6%**	**N=44,588**
breast carcinoma, NOS	25.5%	84.6%	7.7%	7.7%	51
breast invasive ductal carcinoma (IDC)	23.5%	87.5%	12.5%	0.0%	34
breast invasive lobular carcinoma (ILC)	15.0%	66.7%	33.3%	0.0%	20
breast metaplastic carcinoma	13.7%	83.1%	12.3%	4.6%	15,398
breast phyllodes tumor	12.4%	70.0%	23.8%	6.2%	25,864
breast mucinous carcinoma	12.3%	22.7%	71.2%	6.1%	2,555
breast ductal carcinoma in situ (DCIS)	9.1%	0.0%	100.0%	0.0%	11
breast inflammatory carcinoma	7.7%	0.0%	100.0%	0.0%	13
breast papillary carcinoma	3.4%	82.4%	17.6%	0.0%	507
breast lobular carcinoma in situ	0.9%	100.0%	0.0%	0.0%	111
breast carcinosarcoma	0.0%	-	-	-	14
breast myoepithelial carcinoma	0.0%	-	-	-	8
breast adenomyoepithelioma	0.0%	-	-	-	2
**CRC**	**5.7%**	**59.5%**	**35.0%**	**5.5%**	**N=53,322**
colon adenocarcinoma	12.5%	50.0%	50.0%	0.0%	16
rectum adenocarcinoma	6.6%	52.5%	40.6%	7.0%	9,498
rectum squamous cell carcinoma (SCC)	5.5%	61.3%	33.6%	5.1%	43,645
colon adenosquamous carcinoma	1.8%	100.0%	0.0%	0.0%	163
**Bladder**	**18.0%**	**50.8%**	**39.4%**	**9.8%**	**N=9,686**
bladder urothelial (transitional cell) carcinoma	19.0%	51.1%	39.4%	9.5%	8,552
bladder carcinoma, NOS	15.3%	41.2%	40.0%	18.8%	555
bladder squamous cell carcinoma (SCC)	8.1%	69.6%	30.4%	0.0%	283
bladder adenocarcinoma	4.7%	50.0%	42.9%	7.1%	296
**GEC**	**18.1%**	**81.0%**	**14.0%**	**5.0%**	**N=19,657**
esophagus adenocarcinoma	24.4%	85.1%	8.8%	6.1%	7,612
stomach adenocarcinoma, NOS	21.8%	86.3%	9.5%	4.2%	2,074
gastroesophageal junction adenocarcinoma	19.2%	80.0%	10.0%	10.0%	52
esophagus squamous cell carcinoma (SCC)	18.0%	78.4%	13.5%	8.1%	205
esophagus carcinoma, NOS	14.7%	71.8%	24.3%	3.8%	6,763
stomach adenocarcinoma diffuse type	10.7%	80.4%	14.3%	5.4%	524
stomach adenocarcinoma intestinal type	8.7%	40.5%	57.1%	2.4%	481
esophagus adenosquamous carcinoma	5.6%	88.1%	11.9%	0.0%	1,946

CRC, Colorectal Cancer; GEC, Gastroesophageal Cancer; NOS, Not Otherwise Specified; NSCLC, Non-Small Cell Lung Cancer
