# Supplementary material for: Diverse ERBB2/ERBB3 Activating Alterations and Coalterations Have Implications for HER2/3-Targeted Therapies across Solid Tumors
Source: Cancer Res Commun. 2025 Apr 25;5(4):680–93. doi: 10.1158/2767-9764.CRC-24-0620 (PMC12022956; doi:10.1158/2767-9764.CRC-24-0620)
Supplement: Supplementary Table S2 — Histology Breakdown Of Top 5 ERBB3 MUT Cancer Types [file crc-24-0620_supplementary_table_s2_suppst2.pdf]

Supplementary Table S2. Histology Breakdown Of Top 5 *ERBB3* MUT Cancer Types

		% ERBB3 ALT			
**HISTOLOGY**	**ERBB3 ALT PREVALENCE**	AMP	MUT	MULTIPLE	**N**
**CRC**	**2.0%**	**4.8%**	**95.0%**	**0.2%**	**N=53,322**
colon adenosquamous carcinoma	6.3%	0.0%	100.0%	0.0%	16
rectum adenocarcinoma	2.4%	4.3%	95.2%	0.4%	9,498
colon adenocarcinoma	1.9%	4.9%	95.0%	0.1%	43,645
rectum squamous cell carcinoma (SCC)	0.0%	-	-	-	163
**Breast**	**1.7%**	**31.6%**	**67.8%**	**0.5%**	**N=44,588**
breast ductal carcinoma in situ (DCIS)	5.9%	0.0%	100.0%	0.0%	34
breast invasive lobular carcinoma (ILC)	3.0%	7.9%	92.1%	0.0%	2,555
breast mucinous carcinoma	2.0%	0.0%	100.0%	0.0%	51
breast carcinoma, NOS	1.6%	32.7%	66.8%	0.5%	25,864
breast invasive ductal carcinoma (IDC)	1.6%	37.9%	61.3%	0.8%	15,398
breast metaplastic carcinoma	0.4%	0.0%	100.0%	0.0%	507
breast phyllodes tumor	0.0%	-	-	-	111
breast inflammatory carcinoma	0.0%	-	-	-	20
breast papillary carcinoma	0.0%	-	-	-	14
breast lobular carcinoma in situ	0.0%	-	-	-	13
breast carcinosarcoma	0.0%	-	-	-	11
breast myoepithelial carcinoma	0.0%	-	-	-	8
breast adenomyoepithelioma	0.0%	-	-	-	2
**Bladder**	**6.1%**	**14.3%**	**84.8%**	**0.9%**	**N=,9686**
bladder urothelial (transitional cell) carcinoma	6.5%	14.3%	84.8%	0.9%	8,552
bladder carcinoma, NOS	4.9%	18.5%	81.5%	0.0%	555
bladder adenocarcinoma	1.8%	0.0%	100.0%	0.0%	283
bladder squamous cell carcinoma (SCC)	1.0%	0.0%	100.0%	0.0%	296
**GEC**	**4.1%**	**44.3%**	**55.0%**	**0.7%**	**N=19,657**
esophagus adenosquamous carcinoma	5.8%	66.7%	33.3%	0.0%	52
gastroesophageal junction adenocarcinoma	5.0%	61.5%	37.5%	1.0%	2,074
stomach adenocarcinoma diffuse type	4.8%	4.3%	95.7%	0.0%	481
stomach adenocarcinoma, NOS	4.8%	31.9%	68.1%	0.0%	6,763
stomach adenocarcinoma intestinal type	4.4%	22.2%	77.8%	0.0%	205
esophagus adenocarcinoma	4.3%	53.5%	45.0%	1.5%	7,612
esophagus carcinoma, NOS	1.9%	60.0%	40.0%	0.0%	524
esophagus squamous cell carcinoma (SCC)	0.6%	50.0%	50.0%	0.0%	1,946
**Uterine**	**4.3%**	**49.8%**	**48.7%**	**1.5%**	**N=15,631**
uterus adenosarcoma	11.1%	100.0%	0.0%	0.0%	18
uterus endometrial adenocarcinoma papillary serous	5.8%	69.0%	28.4%	2.5%	3,399
uterus endometrial adenocarcinoma mixed histology	5.0%	44.4%	50.0%	5.6%	360
uterus endometrial adenocarcinoma clear cell	4.9%	62.1%	37.9%	0.0%	592
uterus carcinosarcoma	4.6%	67.4%	32.6%	0.0%	2,059
uterus endometrial adenocarcinoma, NOS	4.2%	37.8%	60.9%	1.3%	5,349
uterus endometrial adenocarcinoma endometrioid	2.7%	19.4%	79.6%	1.0%	3,824
uterus endometrial squamous cell carcinoma (SCC)	0.0%	-	-	-	23
uterus perivascular epithelioid cell tumor (PECOMA)	0.0%	-	-	-	4
uterus tumor resembling ovarian sex cord tumors (UTROSCT)	0.0%	-	-	-	3

CRC, Colorectal Cancer; GEC, Gastroesophageal Cancer; NOS, Not Otherwise Specified
