# Supplementary material for: Diverse ERBB2/ERBB3 Activating Alterations and Coalterations Have Implications for HER2/3-Targeted Therapies across Solid Tumors
Source: Cancer Res Commun. 2025 Apr 25;5(4):680–93. doi: 10.1158/2767-9764.CRC-24-0620 (PMC12022956; doi:10.1158/2767-9764.CRC-24-0620)
Supplement: Supplementary Data — SUPPLEMENTARY MATERIALS & METHODS Foundation Medicine Comprehensive Genomic Profiling [file crc-24-0620_supplementary_data_suppsmm.pdf]

SUPPLEMENTARY MATERIALS & METHODS

Foundation Medicine Comprehensive Genomic Profiling

FoundationOne® CDx (F1CDx®) results were analyzed for mutations (single base substitutions and short insertions and deletions), copy number changes (amplifications and homozygous deletions), and large genomic rearrangements, as well as complex biomarkers including microsatellite instability (MSI) and tumor mutational burden (TMB). MSI was calculated by analyzing intronic homopolymer repeat loci for length variability and compiled into an overall MSI score via principal component analysis[1,2]. TMB was calculated across a 0.8-1.2 megabase (Mb) region by counting the number of somatic non-driver synonymous/non-synonymous mutations, a method previously validated against whole exome sequencing[3]. For both F1CDx and FoundationOne®Liquid CDx (F1LCDx™), statistical copy number models were generated and fitted to each individual sample to determine gene copy number. *ERBB2* amplification was defined as gene copy number ≥ sample ploidy +3, while amplification of other genes was defined as copy number ≥ sample ploidy +4. The amplification ratio of the modeled gene copy number to sample ploidy was calculated to quantify copy number amplifications.

Oncogenic/likely oncogenic alterations were called using a multi-step method leveraging annotations which include reporting in COSMIC (RRID:SCR_002260), functional knowledge of the gene affected, internal insights, and clinical/functional characterization in the literature, as described previously[4–6]. Genomic ancestry was predicted based on analysis of single nucleotide polymorphisms (SNPs) trained on data from the 1000 Genomes Project (RRID:SCR_006828) to classify patients as belonging to one of the following subpopulations: African, East Asian, European, South Asian, and Admixed American[7,8]. Clinical features (e.g., cancer diagnosis, age at biopsy collection, HER2 immunohistochemistry [IHC] results, fluorescence *in situ* hybridization [FISH]) results were extracted from test requisition forms and pathology reports.

SUPPLEMENTARY REFERENCES

1. Trabucco SE, Gowen K, Maund SL, Sanford E, Fabrizio DA, Hall MJ, et al. A Novel Next-Generation Sequencing Approach to Detecting Microsatellite Instability and Pan-Tumor Characterization of 1000 Microsatellite Instability–High Cases in 67,000 Patient Samples. J Mol Diagnostics. 2019;21:1053–66.

2. Lin DI, Quintanilha JCF, Danziger N, Lang L, Levitan D, Hayne C, et al. Pan-tumor validation of a NGS fraction-based MSI analysis as a predictor of response to Pembrolizumab. npj Precis Oncol. 2024;8:204.

3. Chalmers ZR, Connelly CF, Fabrizio D, Gay L, Ali SM, Ennis R, et al. Analysis of 100,000 human cancer genomes reveals the landscape of tumor mutational burden. Genome Med. 2017;9:34.

4. Frampton GM, Fichtenholtz A, Otto GA, Wang K, Downing SR, He J, et al. Development and validation of a clinical cancer genomic profiling test based on massively parallel DNA sequencing. Nat Biotechnol. 2013;31:1023–31.

5. Milbury CA, Creeden J, Yip W-K, Smith DL, Pattani V, Maxwell K, et al. Clinical and analytical validation of FoundationOne®CDx, a comprehensive genomic profiling assay for solid tumors. Plos One. 2022;17:e0264138.

6. Lee JK, Sivakumar S, Schrock AB, Madison R, Fabrizio D, Gjoerup O, et al. Comprehensive pan-cancer genomic landscape of KRAS altered cancers and real-world outcomes in solid tumors. Npj Precis Oncol. 2022;6:91.

7. Newberg J, Connelly C, Frampton G. Abstract 1599: Determining patient ancestry based on targeted tumor comprehensive genomic profiling. Epidemiology. 2019;1599–1599.

8. Carrot-Zhang J, Chambwe N, Damrauer JS, Knijnenburg TA, Robertson AG, Yau C, et al. Comprehensive Analysis of Genetic Ancestry and Its Molecular Correlates in Cancer. Cancer Cell. 2020;37:639-654.e6.
